# Genetic, structural, and chemical insights into the dual function of GRASP55 in germ cell Golgi remodeling and JAM-C polarized localization during spermatogenesis

**DOI:** 10.1371/journal.pgen.1006803

**Published:** 2017-06-15

**Authors:** Amandine Cartier-Michaud, Anne-Laure Bailly, Stéphane Betzi, Xiaoli Shi, Jean-Claude Lissitzky, Ana Zarubica, Arnauld Sergé, Philippe Roche, Adrien Lugari, Véronique Hamon, Florence Bardin, Carine Derviaux, Frédérique Lembo, Stéphane Audebert, Sylvie Marchetto, Bénédicte Durand, Jean-Paul Borg, Ning Shi, Xavier Morelli, Michel Aurrand-Lions

**Affiliations:** 1 Aix Marseille Univ, CNRS, INSERM, Institut Paoli-Calmettes, CRCM, Marseille, France; 2 State Key Laboratory of Structural Chemistry, Fujian Institute of Research on the Structure of Matter, Chinese Academy of Sciences, Fuzhou, China; 3 Univ Lyon, Université Claude Bernard Lyon 1, CNRS UMR 5310, INSERM U1217, Institut NeuroMyoGène, Lyon, France; University of Nevada School of Medicine, UNITED STATES

## Abstract

Spermatogenesis is a dynamic process that is regulated by adhesive interactions between germ and Sertoli cells. Germ cells express the Junctional Adhesion Molecule-C (JAM-C, encoded by *Jam3*), which localizes to germ/Sertoli cell contacts. JAM-C is involved in germ cell polarity and acrosome formation. Using a proteomic approach, we demonstrated that JAM-C interacted with the Golgi reassembly stacking protein of 55 kDa (GRASP55, encoded by *Gorasp2*) in developing germ cells. Generation and study of *Gorasp2*^-/-^ mice revealed that knock-out mice suffered from spermatogenesis defects. Acrosome formation and polarized localization of JAM-C in spermatids were altered in *Gorasp2*^*-/-*^ mice. In addition, Golgi morphology of spermatocytes was disturbed in *Gorasp2*^*-/-*^ mice. Crystal structures of GRASP55 in complex with JAM-C or JAM-B revealed that GRASP55 interacted via PDZ-mediated interactions with JAMs and induced a conformational change in GRASP55 with respect of its free conformation. An *in silico* pharmacophore approach identified a chemical compound called Graspin that inhibited PDZ-mediated interactions of GRASP55 with JAMs. Treatment of mice with Graspin hampered the polarized localization of JAM-C in spermatids, induced the premature release of spermatids and affected the Golgi morphology of meiotic spermatocytes.

## Author summary

Spermatogenesis defects are a common cause of male sterility. Spermatogenesis occurs in the seminiferous tubules of the testes and involves adhesive interactions between developing germ cells and Sertoli cells. Knock-out mouse models identified several adhesion molecules that are critically involved in spermatogenesis. We previously demonstrated that the Junctional Adhesion Molecule-C (JAM-C) plays a crucial role in establishing spermatids polarity. The latter involves rearrangements of the Golgi apparatus in spermatids which contribute to acrosome formation. The present study demonstrated that the C-terminal cytosolic region of JAM-C interacted with the Golgi reassembly stacking protein of 55 kDa (GRASP55) encoded by *Gorasp2* and that spermatogenesis was impaired in *Gorasp2*-deficient mice. We developed an inhibitor of GRASP55 interaction with JAM-C and demonstrated that treatment of wild-type mice with the inhibitory compound induced germ cell loss. Therefore, the male infertility-associated pathway identified in this study is important not only from a genetic point of view, but also as a potential target for male contraception.

## Introduction

Members of the Junctional Adhesion Molecular family exhibit a similar structure with two extracellular immunoglobulin domains, a single transmembrane region and a C-terminal PSD-95/Discs Large/ZO-1 (PDZ)-binding motif. Three of these proteins are highly similar: JAM-A, JAM-B and JAM-C [1]. The latter interacts with JAM-B and the leukocyte integrins αMβ2 and αXβ2 [2, 3]. Since JAM-B and JAM-C are both expressed by endothelial cells, it has been proposed that their primary function consists in the regulation of inter-endothelial junctional tightness and leukocyte trans-endothelial migration [4]. However, studies of constitutive and conditional knock-out mice for *Jam3* (the gene encoding JAM-C) revealed an essential function for JAM-C in spermatogenesis [5, 6].

Spermatogenesis occurs in a stepwise manner, beginning with diploid spermatogonia at the basal surface of seminiferous tubules and ending with mature elongated spermatozoa in tubule lumens which are released at spermiation. Spermatogenesis involves adhesive interactions between developing germ and Sertoli cells [7] and is a continuous process that requires 34.5 days in mice. During that time, mitosis, meiosis and maturation occur in spermatogonia, spermatocytes and spermatids, respectively [8, 9]. Spermatogenesis is a developmental system in which the Golgi apparatus undergoes dramatic rearrangements during the meiotic and post-meiotic phases [10]. Germ cells express JAM-C which participates to spermatogenesis via interaction with JAM-B during post-meiotic maturation of spermatids [6, 11]. The strong decrease in sperm cells number in *Jam3*-deficient mice was attributed to the lack of JAM-C recruitment to the junctional plaques at germ/Sertoli cell contacts [6]. Junctional plaques are specialized adhesion structures that anchor germ cells to Sertoli cells and provide spermatids with polarization cues, including JAM-C-mediated polarity signals. The progressive confinement of JAM-C to junctional plaques begins in round spermatids and it is completed in heads of elongated spermatids that remain attached to Sertoli cells via an adhesive structure called apical ectoplasmic specialization [12]. However, little is known about the molecular mechanisms involved in JAM-C polarized localization to spermatids/Sertoli cell contacts. The present study used a combination of proteomic and genetic techniques with structural biochemistry and structure-based drug design approaches to investigate these mechanisms.

We demonstrated that GRASP55 interacted with the PDZ-binding motif of JAM-C in testis. GRASP55 is a medial/trans Golgi molecule that is involved in Golgi stacking, Golgi fragmentation during mitosis and the unconventional protein transport triggered by cellular stress [13–18]. The cargo receptor function of GRASP55 was attributed to the interaction of GRASP55 PDZ domains with motifs in the C-terminal part of cargos such as CD8, TGF-α, or CD83 [19–21].

We solved the 3D structure of GRASP55 in the ligand-free form and in complex with two cargos: JAM-C and JAM-B. The structure revealed a large conformational change between the “open/ligand-free” and “closed/cargo-bound” forms. We used a virtual screening strategy that combined high-throughput docking and pharmacophore filtering to identify protein-protein inhibitors of the GRASP55/JAM interaction [22, 23]. The best inhibitor, referred to as Graspin for “GRASP55 INhibitor”, exhibited reasonable affinity and selectivity for inhibition of GRASP55/JAMs interaction. The biological relevance of GRASP55/JAM-C interaction in spermatogenesis was validated using genetic ablation of *Gorasp2* (encoding GRASP55) and chemical inhibition of GRASP55 PDZ-mediated interactions.

## Results

### GRASP55 interacts with junctional adhesion molecules in a PDZ-dependent manner

**Fig 1:**
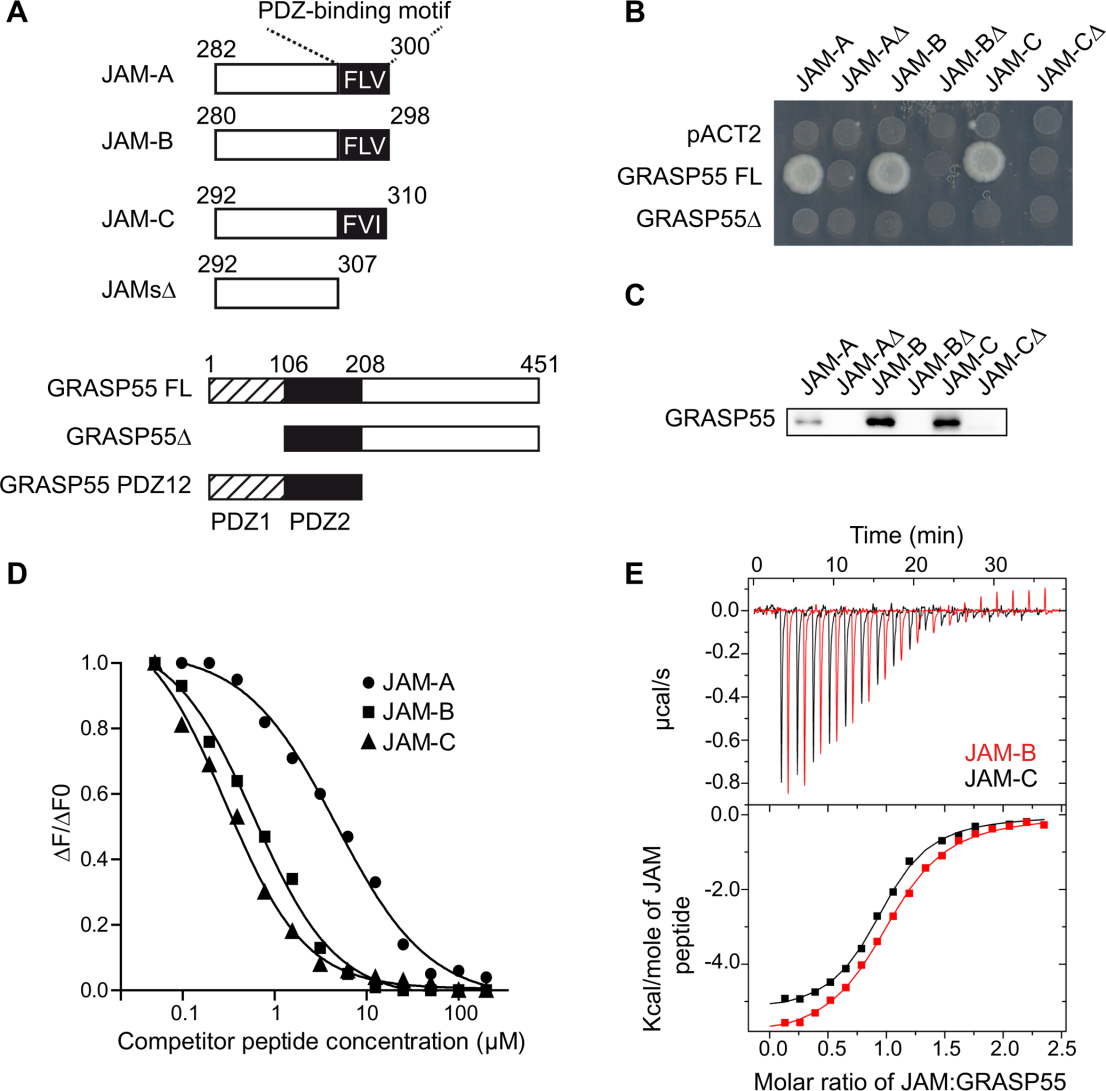
GRASP55 interacts with junctional adhesion molecules in a PDZ-dependent manner. **(A)** Schematic diagram of GRASP55 and JAM-A/B/C constructs. JAMsΔ mutants correspond to wild-type sequences lacking the last three C-terminal amino acids forming PDZ-binding motifs. GRASP55Δ lacks the first PDZ domain and GRASP55 PDZ12 corresponds to the PDZ domain tandem repeat. **(B)** Characterization of GRASP55/JAM interacting domains by yeast two-hybrid using pACT2 as a negative control. **(C)** Immunoblot for GRASP55 after peptide pull-down of testis lysates using the indicated peptides. **(D)** Representative curves obtained by homogenous time-resolved fluorescence (HTRF) using GST-GRASP55 FL and the indicated biotinylated peptides competed with unlabeled peptides. ΔF is calculated as the ratio of signals obtained for acceptor (665nm)/donor (620nm). ΔF0 corresponds to ΔF_max_ obtained in absence of competitor. **(E)** Representative curves obtained by isothermal titration calorimetry (ITC) of GST-GRASP55 FL with JAM-B and JAM-C.

We used a proteomic approach to identify molecular mechanisms that regulate the PDZ-dependent functions of JAMs during spermatogenesis. Testes lysates and peptides corresponding to the terminal 19 amino acids (aa) of JAMs or mutant sequences that lacked the last three C-terminal aa were used in pulldown assays (Fig 1A). Known PDZ-containing binders of JAMs, such as ZO-1 and ZO-2 and, several new binding partners were identified using mass spectrometry (MS), including the Golgi Reassembly Stacking Protein of 55 kDa (GRASP55) (Table in S1 Table). The MS results indicated that the interaction of GRASP55 with JAMs was likely PDZ-dependent because GRASP55 was not pulled-down with the JAM peptides that lacked PDZ-binding motifs. Yeast two-hybrid interaction assays confirmed that the first PDZ domain of GRASP55 was necessary for interaction with JAM proteins (Fig 1B). Conversely, the PDZ-binding motifs of the JAM sequences were required, as demonstrated in the yeast two-hybrid or peptide pull-down assays that were performed with mutant JAM sequences lacking the three C-terminal aa (Fig 1B and 1C). Measurement of the relative binding of GRASP55 to JAM family members using homogenous time-resolved fluorescence (HTRF) or isothermal titration calorimetry (ITC) revealed five- to seven-fold higher affinity interactions of GRASP55 with JAM-B and JAM-C (4.9 μM and 3.7 μM, respectively) as compared to JAM-A (27 μM) (Fig 1D and 1E; Table in S2 Table). Comparable affinities were measured using the full-length GRASP55 protein or isolated tandem PDZ domains (PDZ12) (Table in S2 Table), which supports that the critical residues that contribute to the affinity of GRASP55/JAMs interaction are present within the PDZ tandem domain of GRASP55.

### Impaired spermatogenesis in *Gorasp2*-deficient mice

**Fig 2:**
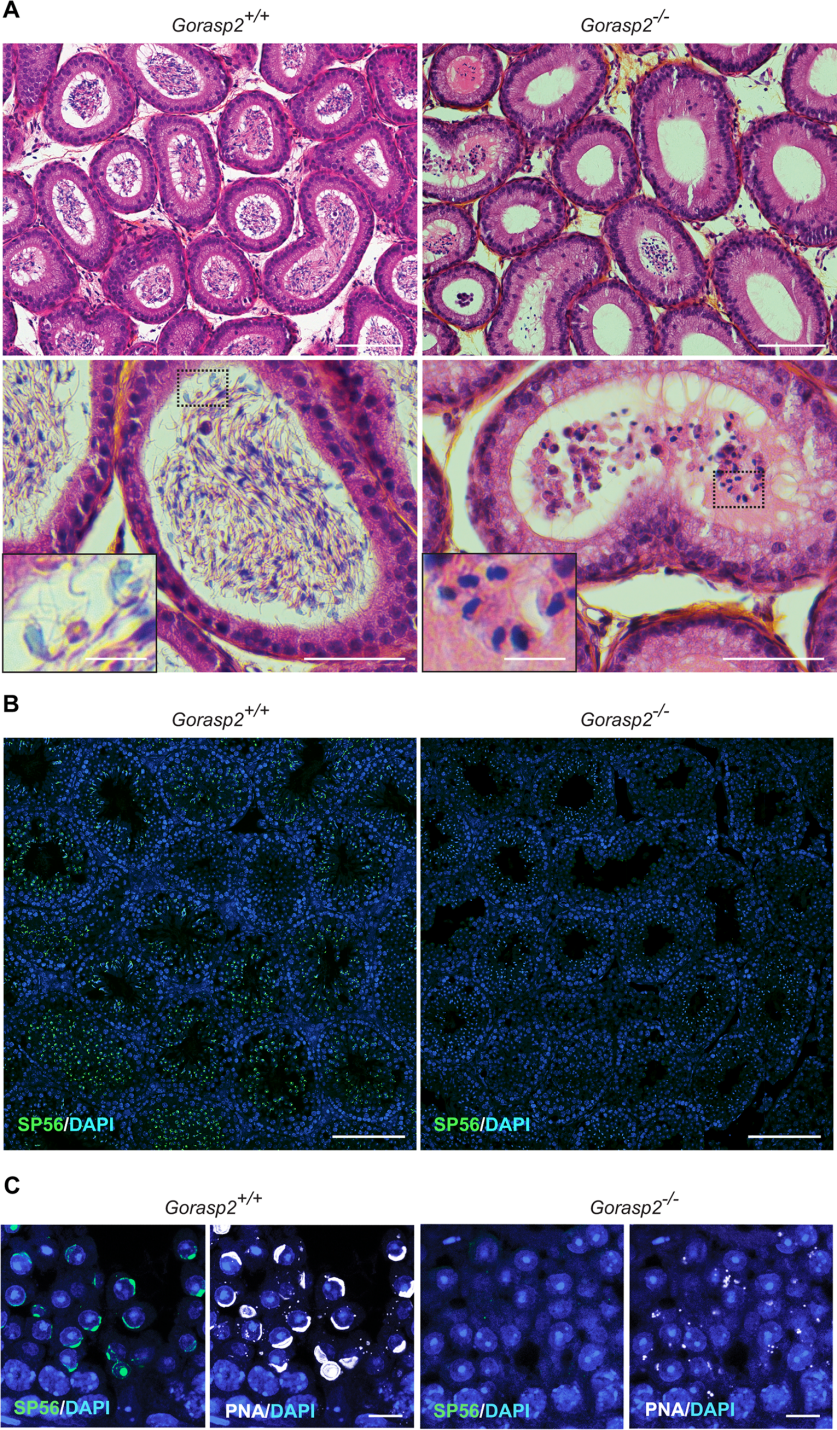
Defective spermatogenesis and acrosome formation in *Gorasp2* mutant mice. **(A)** Sections of epididymis from control littermate and *Gorasp2*^-/-^ mice stained with hematoxylin and eosin. Note that most of the mutant lumens are empty and that some of them contain large round cells instead of differentiated spermatozoa (inserts). Scale bars: top panels, 100 μm; bottom panels, 50 μm; inserts, 10 μm. **(B)** Sections of testes from 35 days-old control and *Gorasp2*^-/-^ mice stained for the acrosomal protein SP56. Scale bar, 100 μm. **(C)** High magnification images of knock-out and control testes sections stained with anti-SP56 and peanut agglutinin (PNA). Note that SP56 staining is more restricted than PNA staining. Scale bar, 10 μm.

**Fig 3:**
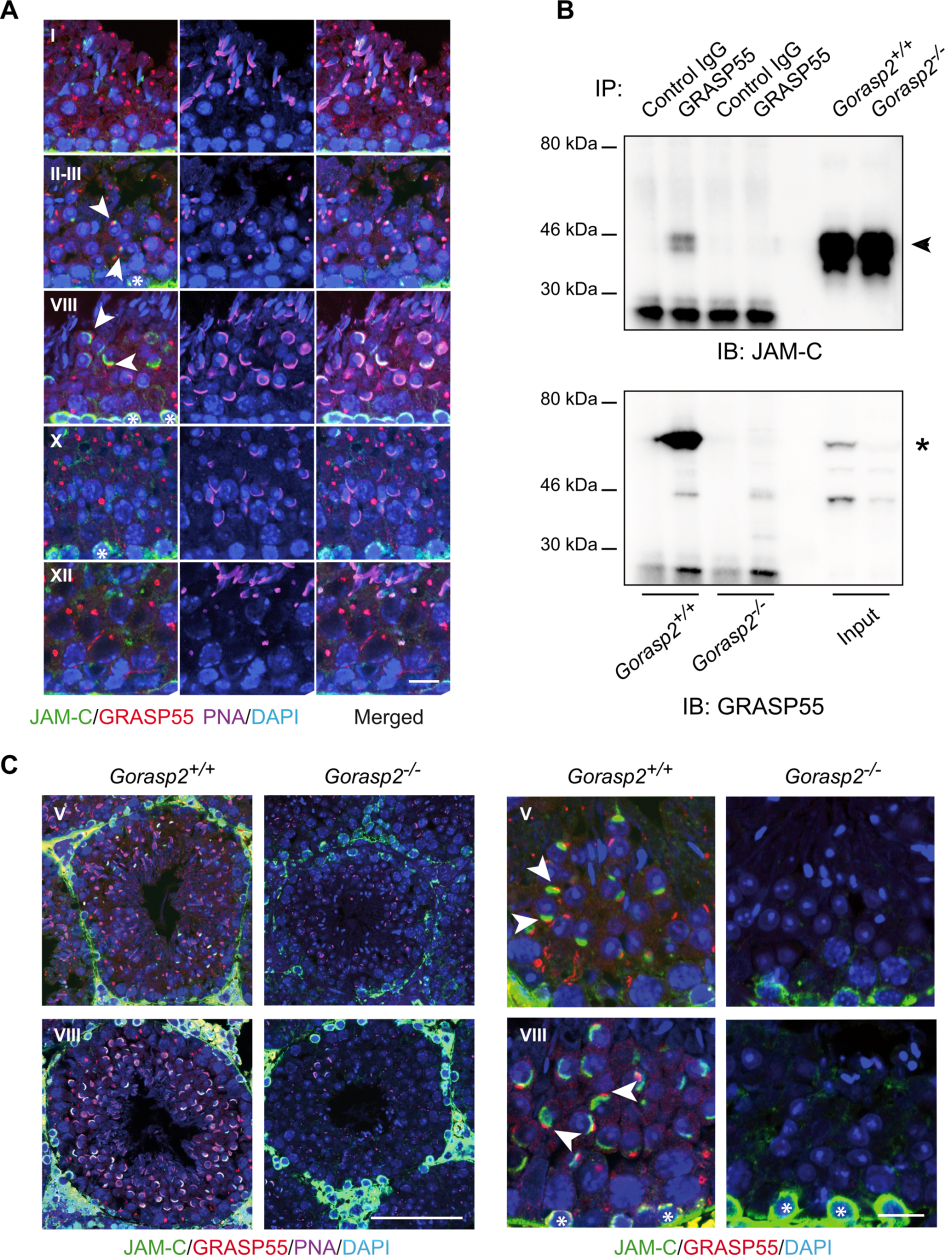
Defective polarized localization of JAM-C in spermatids of *Gorasp2*^*-/-*^ mice. **(A)** Confocal images of GRASP55, JAM-C, peanut agglutinin (PNA) and DAPI staining of seminiferous tubule sections of adult WT mice at stage I, II-III, VIII, X and XII. Staging was performed according to Nakata. H *et al* [26]. Co-polarized distribution of GRASP55 and JAM-C with partial overlap is found in step 2–8 spermatids present in stage II-VIII seminiferous tubules (arrowheads). Note that JAM-C staining overlaps with PNA acrosomal staining in some spermatids. Non-polarized membrane expression of JAM-C is found in spermatogonia (asterisks). Scale bar, 20 μm. **(B)** Co-immunoprecipitation of GRASP55 with JAM-C from testis lysates. Western blots were probed sequentially as indicated. Arrowheads and asterisk indicate the bands corresponding respectively to JAM-C and GRASP55. **(C)** Left panels: Low magnification confocal images of GRASP55, JAM-C, peanut agglutinin (PNA) and DAPI staining of seminiferous tubule sections of adult *Gorasp2*^*+/+*^ and *Gorasp2*^*-/-*^ mice at stage V and VIII. Due to changes in PNA staining, seminiferous tubule staging in knock-out animals was based on morphological criteria. Scale bar: 100 μm. Right panels: High magnification confocal images of GRASP55, JAM-C, and DAPI showing the loss of polarized localization of JAM-C in spermatids of *Gorasp2*^*-/-*^ adult mice as compared to controls (arrowheads). JAM-C expression in spermatogonia is present in *Gorasp2*^*-/-*^ mice (asterisks). Scale bar, 20 μm.

We disrupted the gene encoding GRASP55, *Gorasp2* using homologous recombination to examine the function of GRASP55 *in vivo*, (Fig A-C in S1 Fig). *Gorasp2*-deficient mice exhibited growth retardation, similarly to *Jam3-*deficient mice [24] (Fig D in S1 Fig). Male *Gorasp2*^-/-^ mice bred normally (mating behaviors, plug production), but these mice were infertile. Therefore, we measured number and size of the litters. We never obtained offspring from *Gorasp2*^*-/-*^ males, but *Gorasp2*^*-/-*^ females were fertile (Table 1). Analysis of male reproductive organs isolated from *Gorasp2*-deficient males revealed no significant differences in the testis/body weight ratio or epididymis and seminal vesicles weights (Fig E-G in S1 Fig). However, we observed a trend toward reduced sperm counts isolated from the epididymis (Fig H in S1 Fig). Several defects such as bent midpiece and abnormal head or reduced motility were also found (Fig I-K in S1 Fig). Microscopic examination confirmed that the epididymis of *Gorasp2*^*-/-*^ mice contained rare abnormal cells with large nuclei (Fig 2A), which indicates that spermiogenesis was affected. Spermatid maturation occurs in post-meiotic cells, and it is accompanied by the formation of an acrosome, which is stained with periodic acid-Schiff (PAS) reagent. Light microscopic examination of adult testes from *Gorasp2*^*-/-*^ mice revealed that PAS staining was affected at all tubule stage differentiation, which suggests abnormal acrosome formation (S2 Fig). This result was confirmed using an antibody against a component of the acrosomal matrix, SP56, which becomes detectable at the beginning of acrosome assembly [25]. A complete loss of anti-SP56 staining was observed on testes sections from *Gorasp2*-deficient mice (Fig 2B), and a disorganized and weak residual staining was observed using peanut agglutinin (Fig 2C). These data demonstrated that *Gorasp2* deficiency resulted in acrosomal defects that resembled the spermiogenesis defects previously described in *Jam3*^*-/-*^ mice [6]. Therefore, we examined the relative localization of GRASP55 and JAM-C by immunofluorescence in tissue sections using Tyramide Signal Amplification (TSA) which allows combination of antibodies generated in the same species (i.e. JAM-C and GRASP55 generated in rabbit). This technology is useful, but the enzymatic amplification step hampers comparison of signal intensities between different samples. JAM-C was widely distributed and heavily expressed in spermatogonia and primary spermatocytes. JAM-C expression was reduced in meiotic spermatocytes, with a complete loss in secondary meiotic cells and step 1 spermatids, and weak re-accumulation expression in step 2 spermatids (Fig A, in S3 Fig and Fig 3A). Combination of GRASP55 and PNA staining revealed a co-polarized localization of JAM-C and GRASP55 in step 2 and step 3 round spermatids (Fig 3A, arrowheads). This co-clustering of GRASP55 and JAM-C in the acrosomal region was maintained until stage VIII of seminiferous tubule differentiation, and it was lost in stage X tubules [26]. Co-immunoprecipitation experiments were performed to examine whether a transient interaction between GRASP55 and JAM-C was responsible for the co-polarized localization of these two proteins. Fig 3B shows that the two proteins co-immunoprecipitate. Testes lysates from *Gorasp2*-deficient mice were used as control. We thus questioned if *Gorasp2-*deficiency would affect JAM-C localization to acrosomal region of developing spermatids. Staining revealed that JAM-C expression was strongly reduced in round spermatids at all stages of seminiferous tubule differentiation (Fig 3C), but JAM-C remained expressed in spermatogonia and spermatocytes of *Gorasp2*^-/-^ mice. JAM-B interacts with JAM-C [2] and GRASP55 (Fig 1D and 1E). Therefore, we examined whether JAM-B localization was affected in *Gorasp2*-deficient mice. We found a partial co-localization of JAM-B and JAM-C in spermatocytes and round spermatids in wild-type mice, and this co-localization was lost in *Gorasp2*^-/-^ mice (Fig B in S3 Fig). This result suggests that GRASP55 plays a role in the polarized re-localization of JAM-C with JAM-B at germ/Sertoli cell contacts during spermatid maturation. Spermatid maturation is associated to acrosome formation and apical ectoplasmic specialization assembly. Therefore, testes sections were stained with a well-known marker of apical ectoplasmic specializations, Nectin3 [27]. We observed a complete loss of Nectin3 staining which is consistent with defects in acrosome formation (Fig A in S4 Fig). Other features of seminiferous tubule organization such as JAM-A/ZO-1 localization to basal ectoplasmic specialization or the number of Sertoli cells by seminiferous tubules were not affected in *Gorasp2*^*-/-*^ mice (Fig B-D in S4 Fig), which suggests that the spermatogenic defects in *Gorasp2*^*-/-*^ mice were due to acrosome defects and reduced JAM-C expression in spermatids. “Golgi phase” initiates acrosome formation in step 1 round spermatids and GRASP55 is involved in Golgi apparatus assembly/disassembly [28, 29]. Therefore, we investigated whether *Gorasp2* deficiency also affected the Golgi remodeling that occurs during spermatogenesis.

**Table 1:** GRASP55 genotype and fertility.

**Male**	**Female (+/+)**	**Average number of litters/male over four months**	**Average litter size**
(+/+) n = 4	n = 8	5.75	4.78
(+/-) n = 6	n = 12	5.17	5.28
(-/-) n = 4	n = 8	0	0
Male (+/-)	Female (-/-)	Average number of litters/female over four months	Average litter size
n = 5	n = 10	1.8	4

### *Gorasp2* deficiency affects Golgi morphology in meiotic and somatic cells

**Fig 4:**
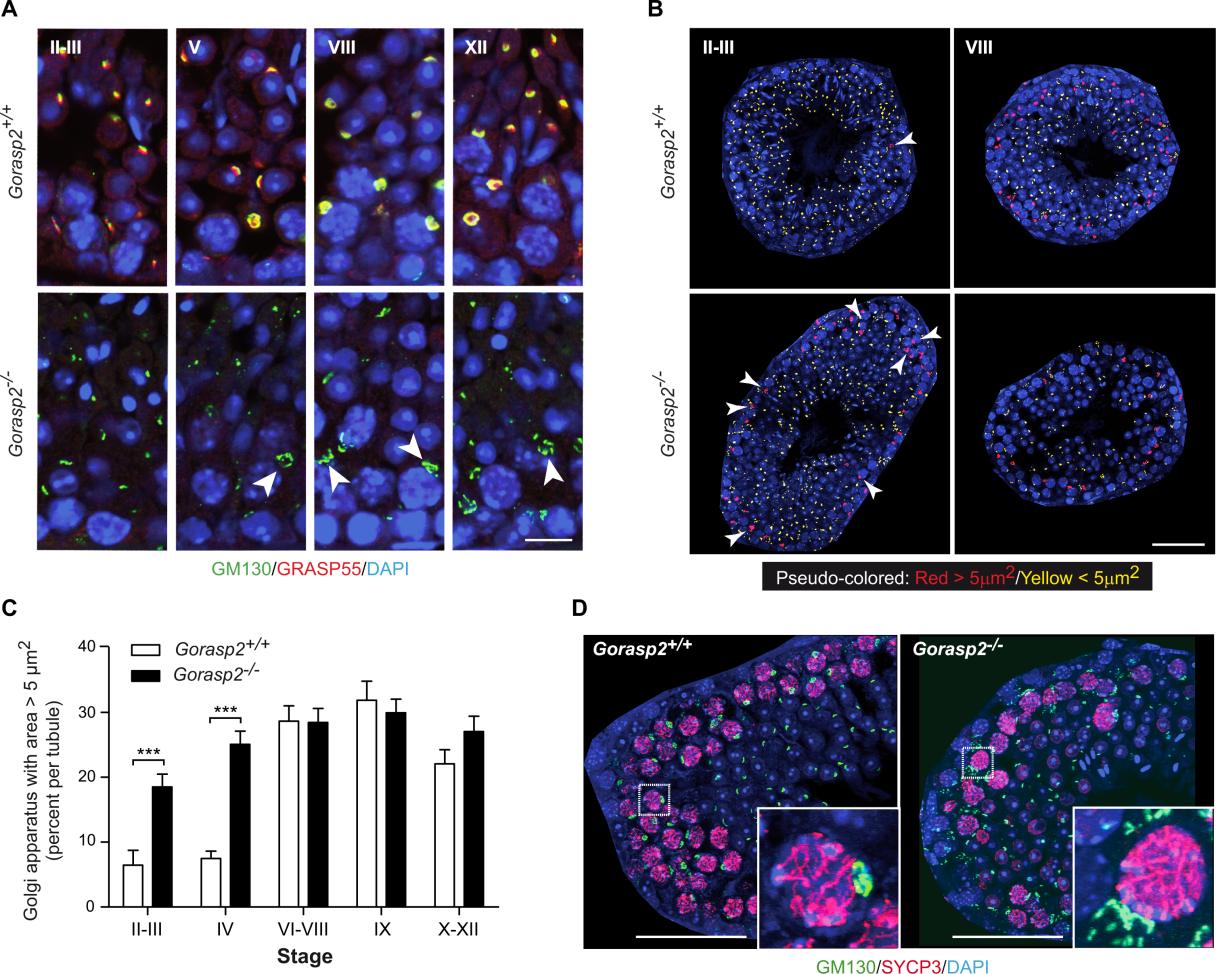
Genetic ablation of *Gorasp2* affects Golgi morphology. **(A)** Confocal images of GM130, GRASP55 and DAPI staining of seminiferous tubule sections of P35 *Gorasp2*^*+/+*^ and *Gorasp2*^*-/-*^ mice at stage II-III, V, VIII and XII. Arrowheads indicate less compact and more diffuse Golgi in spermatocytes from *Gorasp2*^*-/-*^ mice as compared to littermate controls. Scale bar, 10 μm. **(B)** Pseudo-colored images of GM130 staining as a function of Golgi area. The threshold value was set to 5μm^2^ based on analysis of Golgi area distribution in function of seminiferous tubule stages. The automatic pipeline for the functions was encoded in Matlab and is available upon request (arnauld.serge@inserm.fr). Representative pseudo-colored images for indicated tubule stages and genotypes are shown. Arrowheads indicate Golgi with area greater than 5 μm2. Note that Golgi complexes with large areas are essentially localized in the periphery of seminiferous tubules. Scale bar, 50 μm. **(C)** Graph showing the percentages of Golgi with areas greater than 5 μm2 in function of seminiferous tubule differentiation stages. Images used for quantitative analysis were obtained from three independent samples and the number of tubules analyzed was as follow: II-III, n = 27; IV, n = 8; VI-VIII, n = 37; IX, n = 16; X-XII, n = 41. ***: p <0.001. **(D)** Confocal images of GM130, SYCP3 and DAPI staining of seminiferous tubule sections of P35 *Gorasp2*^*+/+*^ and *Gorasp2*^*-/-*^ mice at stage II-III. Scale bar, 50 μm. Inserts: High magnification pictures highlighting GM130 staining in pachytene spermatocytes expressing SYCP3.

We used antibodies directed against the Golgi Matrix protein of 130kD (GM130) to stain testes sections [30]. The results revealed that GM130 staining surrounded GRASP55 signals in spermatocytes of control mice. GM130 staining was more diffuse in spermatocytes from 35-days old *Gorasp2*^*-/-*^ mice compared to littermate controls (Fig 4A, arrowheads). We analyzed Golgi area using GM130 staining in seminiferous tubules at different differentiation stages. We found that few germ cells harbored a Golgi area greater than 5μm^2^ at early stages of seminiferous tubule differentiation (II-III), but cells with an enlarged Golgi area were easily detected at later differentiation stages (VIII) in littermate control mice (Fig 4B). In contrast, we found numerous enlarged Golgi in cells of the early stage tubules of *Gorasp2*^-/-^ mice. Quantification indicated a specific increase in Golgi apparatus with areas greater than 5μm^2^ in stage II-IV seminiferous tubules of *Gorasp2*^-/-^ mice compared to control animals (Fig 4C). Golgi size increases during pachytene spermatocytes maturation prior to separation in four spermatid daughter cells [31]. Therefore, we investigated whether cells with enlarged Golgi corresponded to early spermatocytes using an antibody directed against SYCP3 [32]. Enlarged Golgi in *Gorasp2*^-/-^ mice were present in pachytene spermatocytes of stage II-III seminiferous tubules (Fig 4D). This result indicates that GRASP55 plays a role at an early stage of spermatogenic cell differentiation via regulation of Golgi reassembly at an early stage of meiotic pachytene spermatocyte maturation. We thus tested whether Golgi morphology of somatic cells was also affected by the loss of GRASP55 expression. Primary mouse embryonic fibroblasts (MEFs) isolated from *Gorasp2*-deficient embryos exhibited enlarged Golgi ribbons, which recovered a more compact appearance after GRASP55 re-expression (Fig A in S5 Fig). We developed a dedicated image analysis protocol (Fig B in S5 Fig and Supporting Information) and quantified a two-fold reduction in Golgi density in cells lacking GRASP55 expression. Re-expression of the C-terminal mCherry-tagged form of GRASP55 rescued the Golgi density to the level of wild-type cells (Fig C-D in S5 Fig).

### Co-crystal structure of GRASP55 with JAM-C and JAM-B

**Fig 5:**
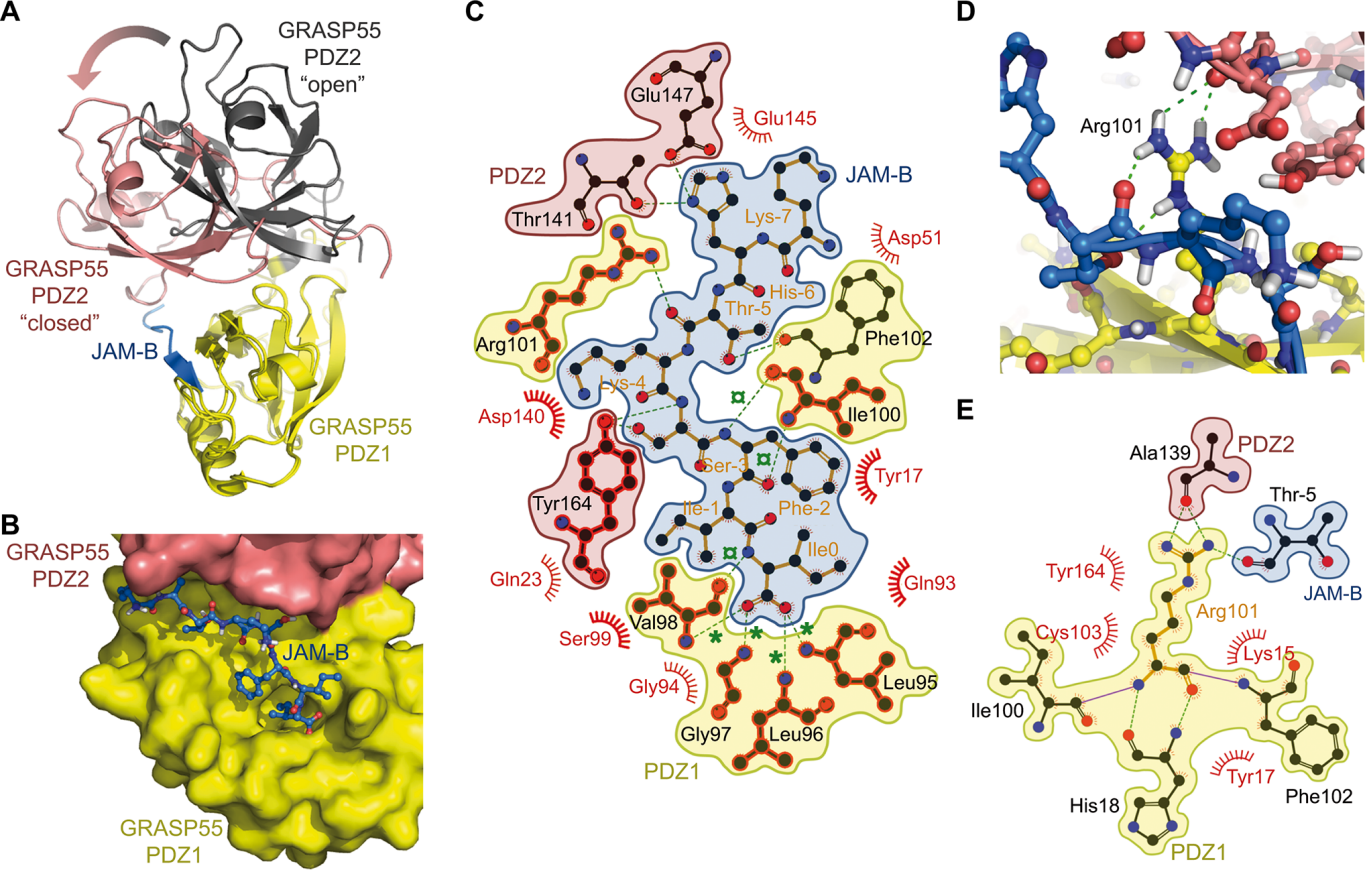
Co-Crystal structure of GRASP55 with JAM peptides. **(A)** Superimposition of the previously reported ‘ligand-free’ GRASP55 PDZ12 X-ray structure (PDB ID: 3RLE) with ‘cargo-bound’ complex, showing GRASP55 PDZ12 structure bound to JAM-B (PDB ID: 5GMI). The ‘ligand free’ published GRASP55 PDZ12 structure is displayed as yellow (PDZ1) and black (PDZ2) ribbons, while the ‘cargo-bound’ conformation is shown as yellow (PDZ1) and salmon (PDZ2); the JAM-B peptide is indicated in blue. The arrow highlights the 33-degree global rotation of the PDZ2 domain after aligning both PDZ1 domains. **(B)** Surface representation of the GRASP55 PDZ12/JAM-B complex structure showing the JAM-B peptide embedded in the groove formed by the GRASP55 PDZ1 (yellow) and PDZ2 (salmon) domains. **(C)** LigPlot+ representation of the complex pinpointing the hydrogen bonding interaction contacts (green) as well as the van der Waals and hydrophobic contacts (semicircles). Green stars (*) highlight carboxylic hydrogen bond contacts in the binding pocket, and green cross circles (¤) indicate β-sheet hydrogen bonding interactions. Conserved contact residues in the JAM-B and JAM-C complex structures are highlighted in red. **(D)** Detailed view of the Arg101 hydrogen bonding interaction network with the PDZ2 domain (salmon) and JAM-B (blue), as revealed by the X-rays structure. **(E)** LigPlot+ representation of the PDZ1 Arg101 hydrogen bonding network with JAM-B and PDZ2. The color coding is the same as that described in (C).

The mode of interaction of GRASP55 with JAMs may aid our understanding of the dual function of GRASP55 in Golgi stacking and JAM-B/JAM-C clustering. Therefore, we co-crystallized GRASP55 PDZ domains with peptides corresponding to the C-terminal 19-mer of mouse JAM-B (JAM-B_P19) and JAM-C (JAM-C_P19). Following the nomenclature for residues binding to PDZ motifs [33], the JAMB_P19 peptide C-terminal Isoleucine residue was designated Ile_0_ and subsequent residues toward the N-terminus were negatively decreased Ile_-1_, Phe_-2_, Ser_-3_, Lys_-4_, Thr_-5_, His_-6_, Lys_-7_ and Phe_-8_. The bound structures of GRASP55 with an uncleaved 6xHis-Tag crystallized in the I4122 space group and contained 2 molecules in the asymmetric unit. The two structures of the complex with JAM-B (PDB ID 5GMJ) or JAM-C (PDB ID 5GMI) were solved at a resolution of 2.99 and 2.71 Å, respectively, using molecular replacement and refined to R_free_ values of 27.4% and 29.1%, respectively (S3 Table). Notably, GRASP55/JAM-C and GRASP55/JAM-B structures exhibited an unexpected ‘closed’ conformation that was characterized by a 33 degree rotation angle of PDZ2 towards the PDZ1 domain and a 12.1 Å root mean square deviation (rmsd) after superimposition of PDZ1 domains to the previously reported structure of the ‘ligand-free’ GRASP55 PDZ domains (Fig 5A) [34]. Normal mode analyses revealed that the transition between the ‘open/ligand-free’ and ‘closed/cargo bound’ conformations was confirmed using the three-lowest frequency normal modes [35, 36], which indicates that both conformations may exist in solution. The cargo bound conformation may be preferentially selected in the presence of C-terminal JAM peptides (Fig A in S6 Fig). These structures indicate that JAM-B_P19 and JAM-C_P19 bind to a groove on the PDZ1 surface, and C-terminal residues penetrate the conventional hydrophobic cavity found in this PDZ domain (Fig 5B; Fig B-C in S6 Fig). Most of the observed interactions occurred via the last four residues of JAM-B_P19 or JAM-C_P19, where the carboxylate group of Ile_0_ is coordinated by a network of hydrogen bonds to the main chain amide groups in the “carboxylate binding loop” of GRASP55 PDZ1 (Fig 5C; Fig D in S6 Fig). This well conserved loop generally exhibits the sequence motif: ϕ-G-ϕ (Leu96-G97-Val98 in GRASP55). Residues at positions 0 and -2 are inserted in an extended conformation and present supplementary hydrogen bonds with the 5^th^ β-strand, which adds a 6^th^ antiparallel β-strand to the conventional structure of the interface. Notably, one very unique feature and non-conventional interaction of GRASP55/JAM-B_P19 was the positioning of Arg101 at a close distance from the interface, which allows hydrogen-bonding interactions with PDZ2 domain amino acids (such as Ala139) and Thr5 from JAM-B (Fig 5D and 5E).

### Identification of a small compound inhibiting GRASP55-PDZ dependent interaction

**Fig 6:**
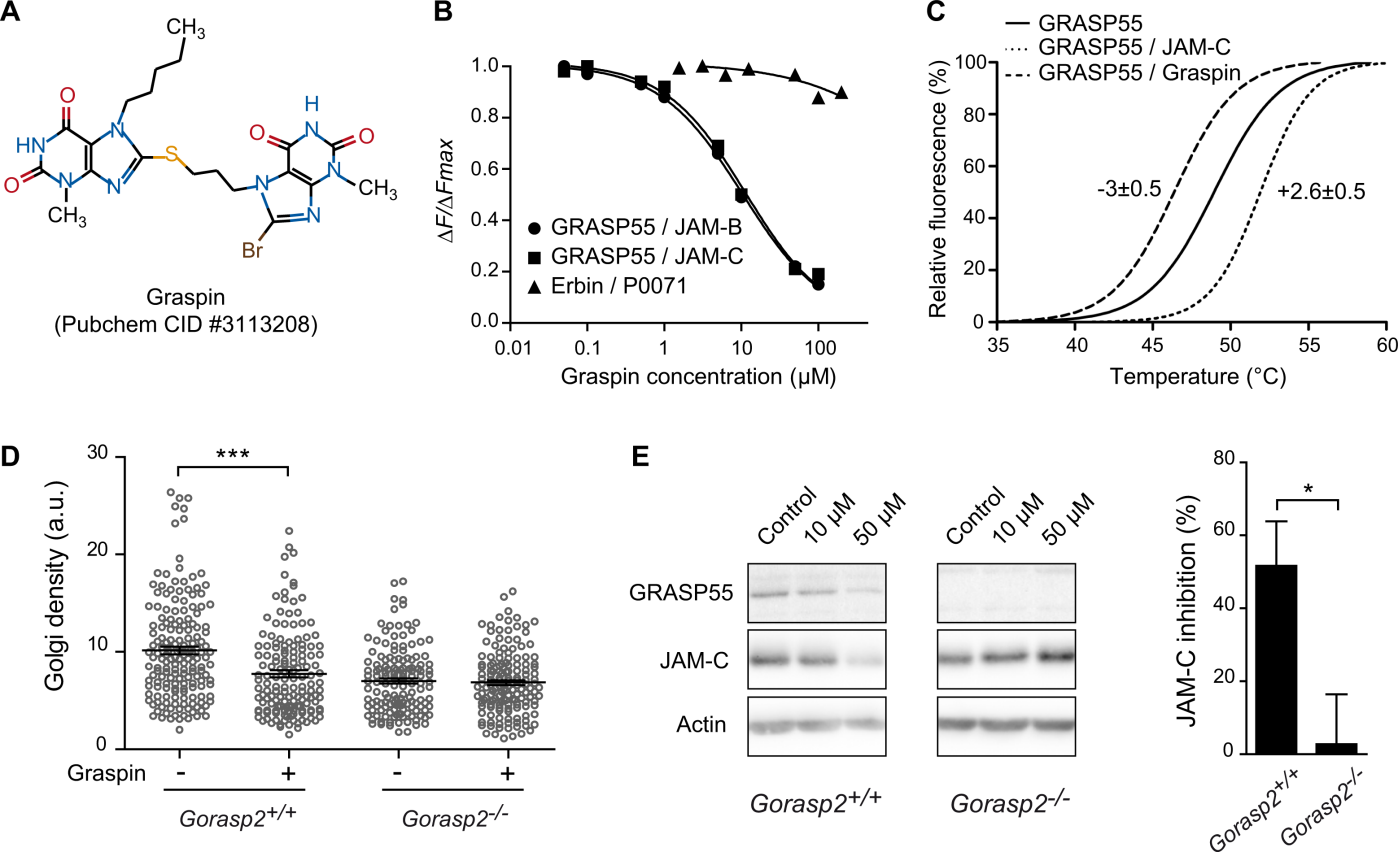
Characterization of a small compound inhibiting GRASP55 interaction with JAMs. **(A)** 2D structure, PubChem Common Identifier (CID) and given name of the prioritized hit from the structure-based drug design approach. **(B)** Representative curves were obtained by HTRF using GST-GRASP55 FL or GST-Erbin with the indicated biotinylated peptides and competed with Graspin (IC_50_ = 8.4 μM for GRASP55/JAM-B and 12 μM for GRASP55/JAM-C). **(C)** Fluorescence profiles presenting results obtained by differential scanning fluorimetry (DSF). Shifts in the melting temperatures of His-GRASP55 PDZ12 incubated with a twelve molar equivalent of ligand or DMSO as control are shown. **(D)** Golgi density of *Gorasp2*^*+/+*^ and *Gorasp2*^*-/-*^ MEFs treated or not with Graspin at 50 μM during 48 h. Each circle represents one Golgi. Data are the mean ± s.e.m. of pooled results of three independent experiments (analysis of 30–90 Golgi per condition, per experiment). Student’s unpaired *t*-test; ***: *P*<0.001. **(E)** Left panel, immunoblot for GRASP55 and JAM-C following exposure of *Gorasp2*^*+/+*^ and *Gorasp2*^*-/-*^ MEFs to increasing doses of Graspin. Right panel, quantification of JAM-C expression in *Gorasp2*^*+/+*^ and *Gorasp2*^*-/-*^ MEFs treated with Graspin at 50 μM for 48 h. Data are expressed as mean of JAM-C inhibition as compared to control condition (vehicle) in five independent experiments. Student’s unpaired *t*-test; *: *P*<0.05.

*In silico* screening for inhibitors of GRASP55/JAM interaction was performed based on the allosteric structural differences between published ‘open/ligand-free’ [34] and ‘closed/cargo bound’ conformations of GRASP55 (this study). The experimental approach was based on a dual strategy using molecular docking and pharmacophore filtering (described in S1 Information). The first step consisted in high-throughput docking of a >200K compounds chemical library dedicated to protein-protein interactions into the binding site of the ‘closed/cargo-bound’ GRASP55 crystal structure (PDB ID 5GMJ). This step was used to generate several conformations that would fit each compound of the chemical library into the binding pocket. The second step filtered million poses using a pharmacophore model. This model was based on the conventional binding interactions observed in the 3D structures of the GRASP55/JAM complex and consisted in 4 hydrogen bond donor/acceptor features and 2 hydrophobic constraints. Several compounds were selected as hits, which were confirmed using orthogonal screening assays. Compound PubChem CID #3113208, referred to as Graspin for “GRASP55 INhibitor” hereafter, exhibited an IC_50_ of 8.4 μM towards GRASP55/JAM-B and 12 μM towards GRASP55/JAM-C as measured by HTRF (Fig 6A and 6B). Graspin did not affect the irrelevant Erbin/P0071 PDZ-mediated interaction. Orthosteric validation using differential scanning fluorimetry (DSF) revealed that, Graspin, but not the JAM-C peptide, decreased the GRASP55 melting temperature (Fig 6C), which suggests that Graspin affected GRASP55 protein stability and should mimic the loss of GRASP55 expression in a biological context. Notably, a reduction in Golgi density in wild-type MEFs was observed after 48 hours of Graspin treatment, but the Golgi density of *Gorasp2*-deficient MEFs was not changed (Fig 6D). We next tested if GRASP55 expression or Graspin treatment affected JAM-C expression or localization in MEFs. No differences in JAM-C expression levels were observed between wild-type and *Gorasp2*-deficient cells in control conditions, but Graspin treatment induced a dose-dependent and specific decrease in GRASP55 and JAM-C expression in wild-type MEFs (Fig 6E). This result indicated that Graspin treatment affected JAM-C expression in a GRASP55 dependent manner, likely due to decreased GRASP55 stability as suggested by the DSF results. In contrast, alternative pathways likely compensate for the constitutive loss of GRASP55 expression in somatic cells to maintain JAM-C expression.

### Graspin alters JAM-C localization to acrosomal region and induces germ cell loss

**Fig 7:**
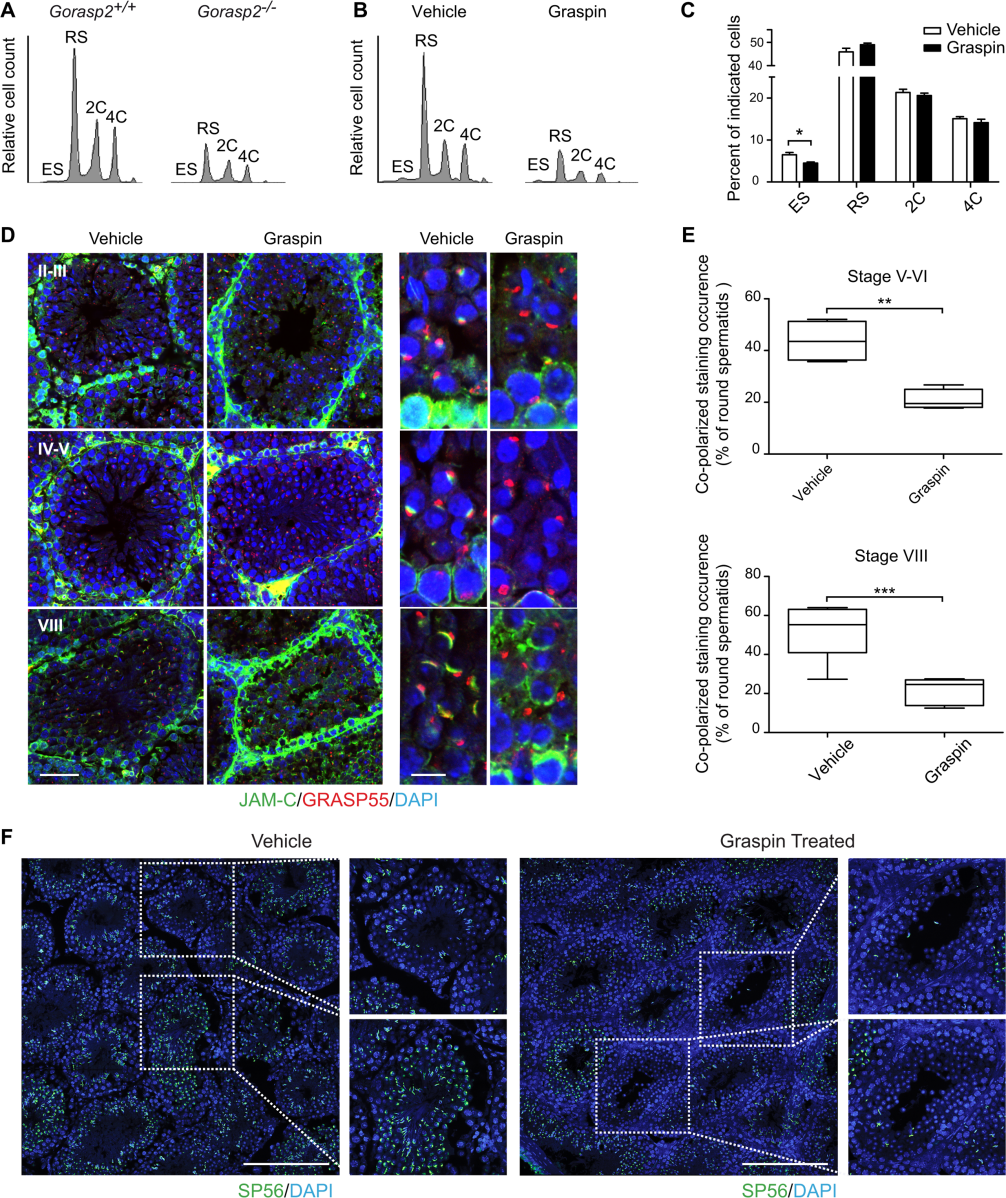
Chemical inhibition of GRASP55 affects spermatid differentiation *in vivo*. **(A-B)** Flow cytometry profiles of DAPI-stained germ cells isolated from testes of *Gorasp2*^*+/+*^ and *Gorasp2*^*-/-*^ 35-days old mice **(A)** and 27-days old mice treated with vehicle or Graspin for two weeks **(B).** ES: elongated spermatids, RS: round spermatids, 2C: spermatogonia, 4C: primary spermatocytes. **(C)** Quantification of germ cell numbers of 27-days old mice treated with Graspin for two weeks and analyzed two days after the last Graspin injection. Vehicle, n = 10; Graspin, n = 10. Student’s unpaired *t*-test; *: *P*<0.05. **(D)** Confocal images of JAM-C, GRASP55 and DAPI staining of seminiferous tubule sections of 43-days old mice treated for 16 days with vehicle or Graspin. Differentiation stages of seminiferous tubules are indicated. High magnification pictures on the left panel highlight the loss of co-polarized localization of GRASP55 and JAM-C in developing round spermatids. Scale bars: left panels: 50 μm; right panels: 10 μm. **(E)** Quantification of co-polarized JAM-C and GRASP55 staining occurrence in control and treated mice expressed as the percentage of round spermatids showing a close apposition between GRASP55 and JAM-C stainings. Results obtained upon quantification of co-polarized staining occurrences in seminiferous tubules at stage V-VI (n = 415 counted events, n = 4 mice, 2 tissue sections/mice) and VIII (n = 498 counted events, n = 6 mice, 2 tissue sections/mice) are shown. Student’s unpaired *t*-test; **: *P*<0.01, ***: *P*<0.001. **(F)** Confocal images of SP56 and DAPI staining of seminiferous tubule sections of 43-days old mice treated for 16 days with vehicle or Graspin as indicated. High magnification pictures on the right panels highlight the loss of SP56 staining associated with elongated spermatids. Scale bars: 200 μm.

**Fig 8:**
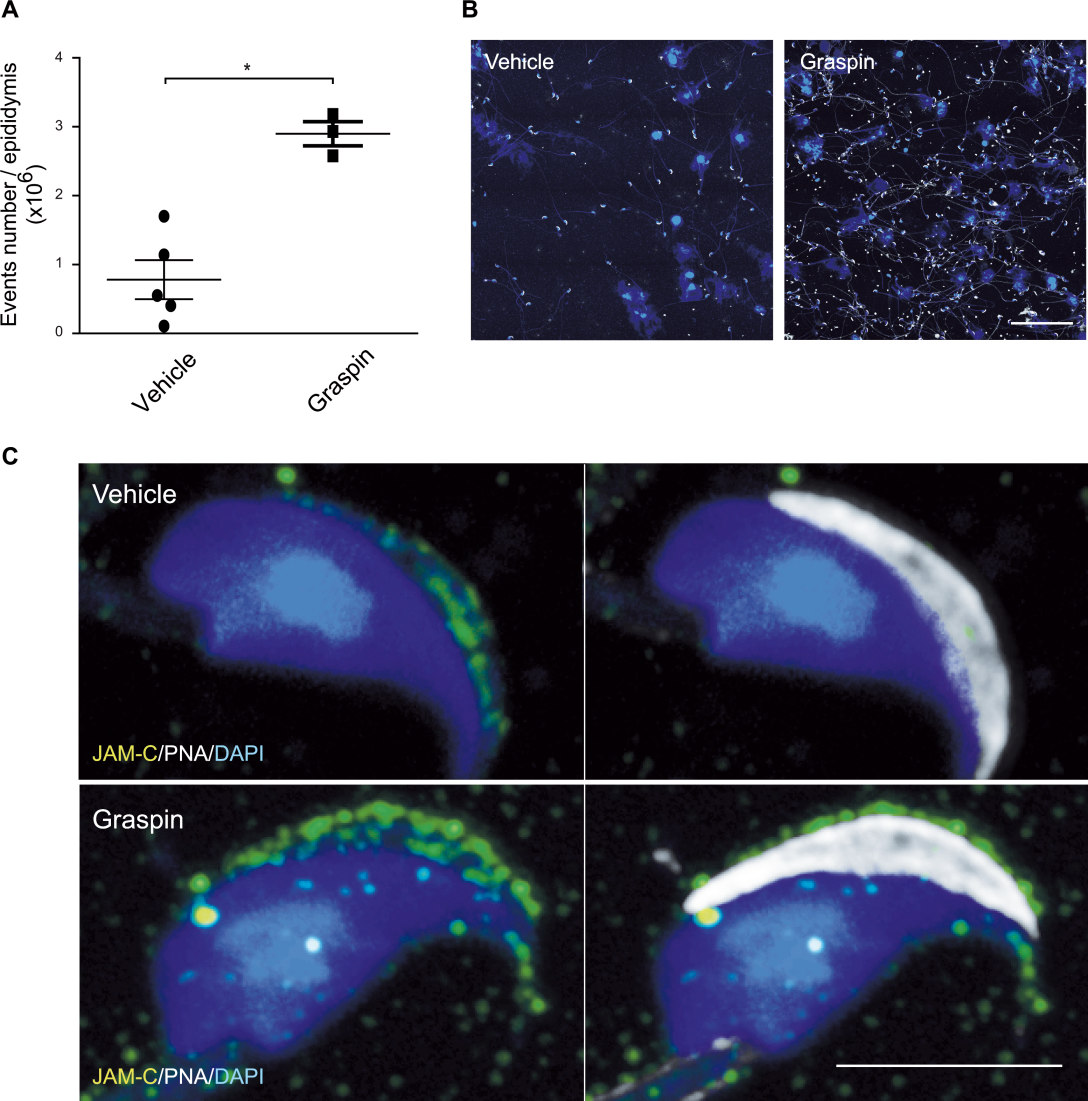
Graspin treatment induces germ cell release in epididymis. **(A)** Quantification of epididymis content from vehicle and Graspin treated mice using flow-cytometry. Student’s unpaired *t*-test; *: *P*<0.05. **(B)** Cytospin of material recovered from epididymis of vehicle and Graspin treated mice and stained for DAPI and PNA. Note the increased abundance and heterogeneity of material recovered from Graspin treated animals. Scale bar, 100μm **(C)** Representative high magnification confocal images of spermatozoa recovered from epididymis of vehicle or Graspin treated mice and stained for DAPI (blue), JAM-C (green) and PNA (grey). Note that the co-localization of JAM-C with PNA is lost in samples from Graspin treated mice. Scale bar, 5μm

**Fig 9:**
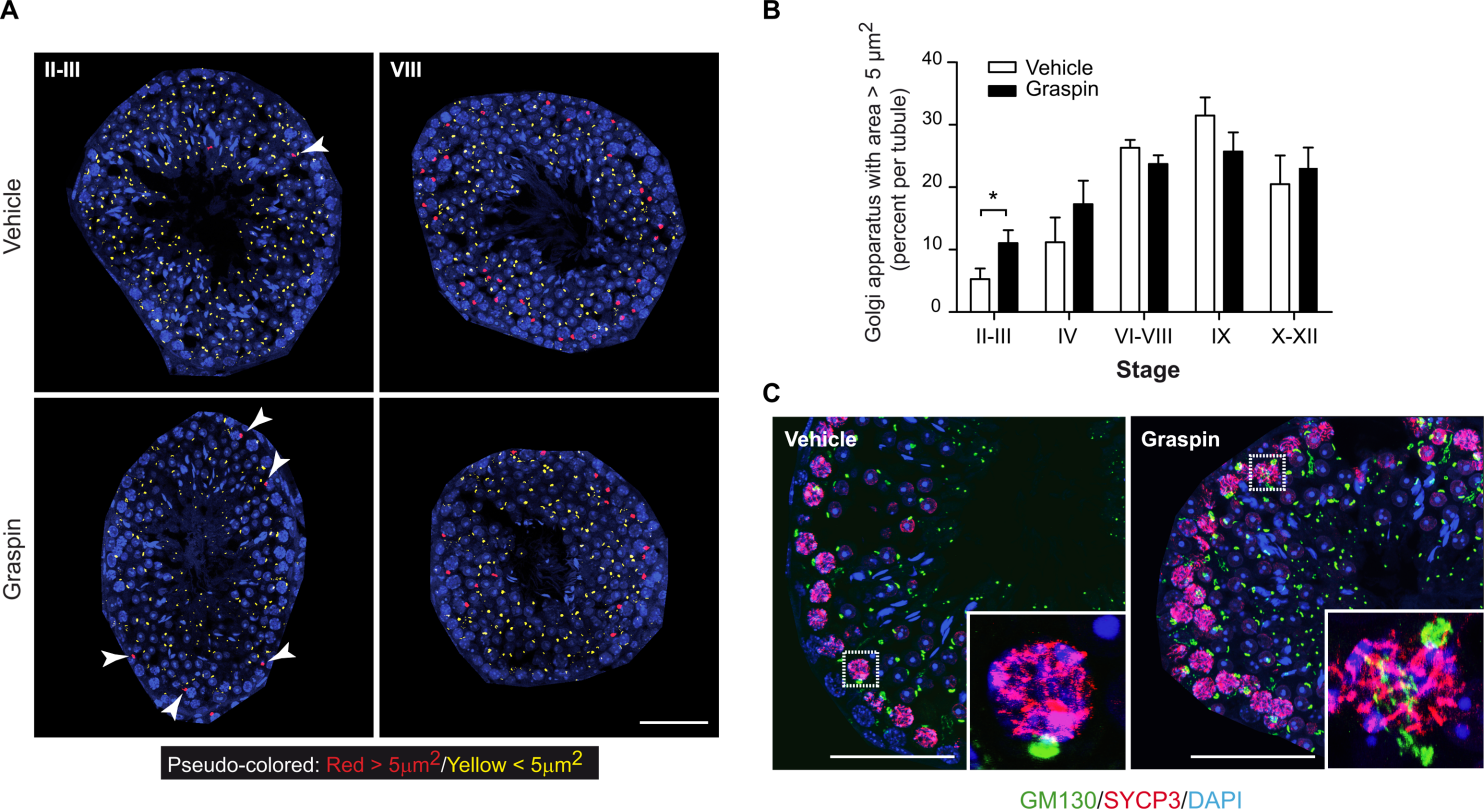
Graspin treatment affects Golgi morphology of germ cells. **(A)** Pseudo-colored images of GM130 staining as a function of Golgi area. The threshold value was set to 5μm^2^ based on analysis of Golgi area distribution in function of seminiferous tubule stages. Representative pseudo-colored images for indicated tubule stages and genotypes are shown. Arrowheads indicate Golgi with area greater than 5 μm2. Note that Golgi with large areas are essentially localized in the periphery of seminiferous tubules. Scale bar, 50 μm. **(B)** Graph showing the percentages of Golgi area greater than 5 μm2 in function of seminiferous tubule differentiation stages. Images used for quantitative analysis were obtained from three independent samples and the number of tubules analyzed was as follow: II-III, n = 30; IV, n = 12; VI-VIII, n = 47; IX, n = 16; X-XII, n = 28. *: p <0.05. **(C)** Confocal images of GM130, SYCP3 and DAPI staining of seminiferous tubule sections of P35 treated mice at stage II-III. Scale bar, 50 μm. Inserts: High magnification pictures highlighting GM130 staining in pachytene spermatocytes expressing SYCP3.

*Gorasp2* deficiency results in spermatogenesis defects and loss of JAM-C localization in the acrosomal region. Therefore, we examined whether Graspin treatment affected spermatogenesis *in vivo*. Treatment was initiated in 27-days old mice to begin experiments in animals that did not experience a single wave of germ cell development, which ends on day 35 (Fig A in S7 Fig). No obvious toxicity or changes in seminiferous tubule composition were observed under these conditions (Fig B-C in S7 Fig). However, obvious tubule disorganization was visualized using DAPI/PNA staining of testes sections (Fig D in S7 Fig), which suggests that tubule content was affected. This result was confirmed using flow cytometry and DAPI staining which allow quantification and discrimination of elongated spermatids (ES), round spermatids (RS, 1C), spermatogonia (2C) and primary spermatocytes (4C) [37]. A marked reduction of all spermatogenic cells was observed in Graspin-treated and *Gorasp2*-deficient mice (Fig 7A and 7B). Quantification of flow-cytometry experiments revealed a specific decrease in the percentage of elongated cells in testes of Graspin-treated mice (Fig 7C). This result is consistent with the twofold decrease in ES content observed on histological sections (Fig E in S7 Fig). The expression of flow-cytometry results as absolute numbers revealed an overall two-fold reduction in testes cellularity (Fig F in S7 Fig). This result suggests that Graspin induced ES depletion and affected spermatogenesis at earlier stage of differentiation, which decreased cellularity. Analysis of testes sections isolated from treated mice and stained for JAM-C and GRASP55 revealed that the down-regulation of JAM-C at the transition between spermatocyte and spermatids and the re-localization of JAM-C in the acrosomal region of RS were severely affected (Fig 7D). Quantification of the frequency of co-polarized GRASP55/JAM-C staining in RS at stage V-VI and stage VIII revealed that the co-clustering of JAM-C staining with GRASP55 was severely decreased with Graspin treatment (Fig 7E). This result prompted us to investigate whether Graspin treatment affected acrosomes. A strong decrease in SP56 staining and obvious reduction in ES content of some tubules was found after Graspin treatment (Fig 7F). These effects were not due to increased apoptosis as revealed by TUNEL staining (S8 Fig), which suggests that it may be due to disruption of apical ectoplasmic specialization and “premature spermiation”, as previously reported for other compounds that affect spermatogenesis [38]. Flow-cytometry comparison of epididymis content of Graspin- and vehicle- treated mice revealed a threefold increase in spermatozoa and cell debris in epididymis of Graspin treated mice (Fig 8A and 8B). This increase was accompanied by a mislocalization of residual JAM-C staining to the acrosome of mature spermatozoa (Fig 8C), which suggests that Graspin impaired the coordinated regulation of apical ectoplasmic specialization via inhibition of GRASP55 PDZ-mediated interactions. Graspin treatment also affected the Golgi density of pachytene spermatocytes which exhibited a significant increase in the frequencies of Golgi with areas greater than 5 μm^2^ in stage II-III tubules (Fig 9). Altogether, our data demonstrate that Graspin treatment mimics *Gorasp2* deficiency and affects spermatogenesis via targeting Golgi reassembly in spermatocytes and inhibition of acrosomal related functions in spermatids.

## Discussion

JAM-C interacts with PAR3 via its PDZ-binding motif and it associates with CRUMBS (CRUMBS3/PALS1/PATJ) and PAR (PAR3/PAR6/aPKC) polarity complexes in spermatids [6, 39]. In addition, JAM-C localizes to the acrosome of spermatozoa isolated from epididymis [40]. The constitutive or conditional deletion of *Jam3* in germ cells results in a loss of cytoskeletal protein polarization with an arrest of differentiation at the stage of round spermatid [6]. The role of JAM-C in germ cell polarity and adhesion to Sertoli cell was further confirmed using a small compound that destabilizes apical ectoplasmic specializations, adjudin [41]. In this study, the authors reported that adjudin-induced germ cell loss was accompanied by a decrease in JAM-C association with PALS1/PAR6, which may contribute to sperm cell release. However, the dynamic localization and trafficking of JAM-C to apical ectoplasmic specialization or acrosome is still poorly understood.

The present study identified GRASP55 as an endogenous interacting partner of the JAM-C PDZ-binding motif in developing germ cells. *Gorasp2*^-/-^ mice display male infertility but do not present other gross morphological defects, similarly to *Jam3*-deficient mice. The major defects of *Gorasp2*^*-/-*^ developing germ cells were defects in acrosome formation, a reduced number of elongated spermatids, a lack of polarized localization of JAM-C in round spermatids and a dramatic enlargement of Golgi apparatus in early pachytene spermatocytes. These results raise the question of how a single Golgi protein can interfere with meiosis, acrosome formation and JAM-C trafficking?

Landmark studies have documented changes in Golgi morphology during meiotic division of spermatocytes or during early spermiogenesis [42, 43]. However, the underlying molecular mechanisms are poorly understood. The Golgi size of rat pachytene spermatocytes increases from a diameter of 0.5–1 μm at stages I-III to 2–3 μm at stages IV-XII of seminiferous cycle [31]. This increase is consistent with our results showing that the threshold value of 5 μm^2^ for Golgi area discriminates between the spermatocytes in early (II-III) and late stage seminiferous tubules (VI-XII). One remarkable finding was that chemical or genetic inhibition of GRASP55 resulted in Golgi enlargement of early pachytene spermatocytes, which suggests a delay of Golgi reassembly in these cells. The pachytene spermatocytes represent the longest phase of prophase during the first meiotic division [9]. Therefore, defects in Golgi reassembly may delay pachytene spermatocytes maturation and decrease cellularity as a consequence of meiotic phase lengthening. These changes are consistent with the known function of GRASP55 in Golgi stacking and breakdown in mitotic somatic cells [16, 44].

Another finding was that chemical inhibition of GRASP55 resulted in defects of acrosome formation and premature spermiation. Acrosome development occurs during early spermiogenesis and results from the assembly of pro-acrosomic vesicles. These vesicles originate from the Golgi apparatus and GRASP55 has been reported to be specifically associated to the Golgi apparatus and acrosome of step 1–7 rat spermatids, which suggests that this protein plays a specific function in acrosome development [45]. Our results confirmed this hypothesis and demonstrated that this function relies, at least partially, on the transient interaction between GRASP55 and JAM-C during early spermiogenesis in mice (step 1–7 spermatids). Therefore, we propose a model in which GRASP55 is involved in the coordinated regulation of JAM-C expression and localization in spermatids that contribute to apical ectoplasmic specialization polarity complex anchoring. Indeed, *Gorasp2* deficiency resulted in acrosomal defects and the subsequent lack of polarized localization of JAM-C in the acrosomal region. Graspin inhibition of GRASP55 PDZ-mediated interactions induced more subtle changes in JAM-C expression and localization and resulted in “premature spermiation”. These effects are similar to what has been described for adjudin, which is a potential male contraceptive that specifically perturbs the function of apical ectoplasmic specializations [38, 46]. Notably, adjudin treatment affects the association of JAM-C with polarity complex proteins [41]. These JAM-C-mediated interactions are PDZ-binding-motif-dependent and should be mutually exclusive from the interaction of JAM-C with GRASP55 [39], which suggests that the JAM-C-interacting PDZ network plays a central role during spermiogenesis.

Finally, our study revisits the structural properties of GRASP55. A previously published, 3D structure of GRASP55 (PDZ12) revealed an unusual metazoan circularly permutated PDZ domain-containing protein in which one PDZ domain contains a unique internal peptide ligand for the second PDZ domain. This intermolecular interaction between GRASP55 proteins was thought to form a strong and stable complex that bridged adjacent molecules and maintained Golgi stacks [34]. GRASP55 interaction with Golgin45 may also contribute to the Golgi stacking function of GRASP55 [47]. These intermolecular interactions between GRASP55 PDZ domains may be disrupted during the post-meiotic transition between spermatocytes and spermatids, and the PDZ1 ligand-binding domain of GRASP55 may be re-affected to JAM-C receptor function. This hypothesis is consistent with a proposed model in which the allosteric regulation of GRASP via phosphorylation disrupts it self-association and leads to Golgi breakdown during mitosis [14, 48, 49]. Our 3D structure pinpoints that the GRASP55/JAM-C (or JAM-B) complex involves a 3D interaction that induces significant conformational changes between the ‘ligand-free’ (‘Golgi bound’ conformation as described by Truschel et *al* [48]) and the ‘cargo-bound’ conformation of the protein (this study). The overlay of our structures with the published ‘ligand free’ form of GRASP55 reveals that the conformational changes occur in the main chain of the second PDZ domain (PDZ2) and its relative orientation to PDZ1, which compacts the PDZ2 into a ‘closed/cargo-bound’ conformation. Most of the interactions are present around the conventional hydrophobic cavity in the PDZ1, but our structures reveal a very unique feature outside the conventional binding mode of the PDZ domain. Several supplementary intramolecular hydrogen bonds involving the Arg101 residue from the PDZ1 and Ala139 from the PDZ2 of GRASP55 were identified and contributed to the conformational exchange between free and bound conformations.

In summary, our findings report the first non-redundant function for GRASP55 in mammals and establish a link between the function of GRASP55 in germ cells and the subcellular localization of JAM-C in spermatids. We also provide evidence that the inhibition of GRASP55 PDZ-mediated interactions using a small compound affects spermatogenesis via reduction of overall cellularity and induction of “premature spermiation”. These results demonstrate that the chemical targeting of PDZ scaffolds involved in complex biological pathway may be achieved *in vivo* which paves the way toward therapeutic targeting of PDZ-mediated interactions.

## Materials and methods

### Antibodies

Rabbit anti-GRASP55 (ref. 10598-1-AP, ProteinTech), rabbit anti-JAM-B 829 [50, 51], rabbit anti-JAM-C 501 [51], goat anti-JAM-C (ref. AF1213, R&D system), mouse anti-GM130 (ref. 610822, BD Biosciences), mouse anti-SP56 (ref. MA1-10866, ThermoFisher Scientific), rabbit anti-Nectin3 (ref. ab63931, Abcam), rabbit anti-SYCP3 (ref. ab15093, Abcam) and mouse anti-actin (ref. A3853, Sigma) primary antibodies and biotinylated PeaNut Agglutinin (PNA, ref. L6135, Sigma) were used for immunostaining and immunoblotting. Appropriate anti-rabbit, anti-goat or anti-mouse secondary antibodies were obtained from Jackson Immuno-research Laboratories.

### Generation of GRASP55-expressing constructs and production of fusion proteins

The full-length *Gorasp2* cDNA encoding the GRASP55 protein was amplified by polymerase chain reaction (PCR) using the oligonucleotides 5'-CTCGAGATGGGCTCCTCGCAGAGC-3' and 5'-GGATCCCCAGAAGGCTCTGAAGCATCTGC-3', containing *Xho*I and *Bam*HI sites, respectively. The amplification product was cloned in the pGEM-T Easy vector (Promega). The insert was recovered by *Xho*I/*Bam*HI digestion and subcloned in the pmCherry-N1 vector (Clontech). To generate fusion proteins, mouse *Gorasp2* cDNA cloned into pGEM-T was used as template for PCR amplification of the open reading frame (ORF) for mGRASP55 PDZ1 (aa 2–107), PDZ12 (aa 2–208), mGRASP55 Full-length (FL) (aa 2–451) or GRASP55Δ (aa 106–451) using forward and reverse oligonucleotides flanked by *attb1* and *attb2* recombination sites. The following primers pairs were used: PDZ1 For: 5'- GGGGACAAGTTTGTACAAAAAAGCAGGCTTCCTGGTTCCGCGTGGATCCGGCTCCTCGCAGAGCGTCG-3’ or 5'-GGGGACAAGTTTGTACAAAAAAGCAGGCTTCGGCTCCTCGCAGAGCGTCGAGAT-3’ (with and without sequence coding for a thrombin cleavage site, respectively); PDZ2 For:

5'-GGGGACAAGTTTGTACAAAAAAGCAGGCTTCGGGGCCAACGAAAACGTTTGGCATGTGCTG-3’

PDZ1 Rev: 5'-GGGGACCACTTTGTACAAGAAAGCTGGGTCTTACCCGTCAAAGCTGCAGAAACGAATGCT-3', PDZ2 Rev: 5'-GGGGACCACTTTGTACAAGAAAGCTGGGTCTTATTCAAAGGGGCGTGTAGGTATTCGGTGCA-3' and GRASP55 FL Rev: 5'-GGGGACCACTTTGTACAAGAAAGCTGGGTCTTAAGAAGGCTCTGAAGCATCTGCATCAGAC-3'. The amplicons were cloned by the BP reaction into pDONRZeo (Gateway® Technology) to produce the corresponding entry vectors. The coding sequences were transferred by LR cloning in pDESTTM15 and pDESTTM17 prokaryotic expression vectors intended to produce the corresponding N-terminal GST- or 6His-tagged fusion protein, respectively. This was accomplished by induction for 3 h at 37°C or 18 h at 25°C with 0.2 mM isopropyl-β-D-thiogalactopyranoside in *E*. *coli* BL21 (DE3) bacteria cells transformed with the purified plasmids. Fusion proteins were recovered from the cell lysates by conventional affinity chromatography on Glutathione sepharose 4B (GE17-0756-01, Sigma-Aldrich) or Ni-NTA Agarose (R90115, ThermoFisher). The 6His-tagged PDZ12 used for crystallography was further purified using Resource Q Sepharose anion exchange (17-1177-01, GE Healthcare) followed by Superdex 75 gel-filtration chromatography (17-5174-01, GE Healthcare).

### Peptide pulldown and mass spectrometry analysis

Biotinylated 19-mer peptides corresponding to the carboxy-terminal sequences of JAM-A (Biotin-SQPSTRSEGEFKQTSSFLV), JAM-B (Biotin-SKVTTMSENDFKHTKSFII), JAM-C (Biotin-NYIRTSEEGDFRHKSSFVI), and the same sequences lacking the three last amino acids (Covalab, France) were immobilized on streptavidin Sepharose high-performance beads (GE Healthcare). Wild-type mouse testes were isolated, frozen in nitrogen, crushed with a pestle and solubilized in lysis buffer (50 mM HEPES pH 7.3, 10% glycerol, 0.1 mM EDTA, 150 mM NaCl, 1% Triton X100 and protease inhibitors). One milliliter of testis lysate (5 mg of protein) was added to the peptide-coupled beads (20 μL) and incubated for 2 h. The beads were washed 5 times in lysis buffer, boiled in Laemmli buffer, and proteins were analyzed by mass spectrometry or immunoblotting. To visualize proteins by silver staining, 10% of the denatured protein extracts were loaded in a 4–12% Bis-Tris gradient pre-cast NuPAGE gel and run with MOPS buffer according to the manufacturer’s instructions (Invitrogen). For mass spectrometry analysis, 90% of the denatured protein extracts were also loaded in a 4–12% Bis-Tris acrylamide gel, but running of the samples was stopped as soon as the proteins had stacked as a single band. Protein-containing bands were stained with Imperial Blue (Thermo Scientific), cut from gel, and following reduction and iodoacetamide alkylation, digested with high sequencing grade trypsin (Promega). The extracted peptides were further concentrated before analysis. Mass spectrometry analysis was conducted by liquid chromatography-tandem mass spectrometry (LC-MSMS) using a LTQ-Velos-Orbitrap (Thermo Scientific) online with a nanoLC RSLC Ultimate 3000 chromatography system (Dionex). Five microliters corresponding to 1/5^th^ of the whole sample was injected into the system in triplicate. After pre-concentrating and washing the sample on a Dionex Acclaim PepMap 100 C18 column (2 cm × 100 μm i.d., 100 Å, 5 μm particle size), the peptides were separated on a Dionex Acclaim PepMap RSLC C18 column (15 cm × 75 μm i.d., 100 Å, 2 μm particle size) at a flow rate of 300 nL/min with a two-step linear gradient (4–20% acetonitrile/H_2_O; 0.1% formic acid for 90 min and 20-45-45% acetonitrile/H_2_O; 0.1% formic acid for 30 min). For peptide ionization using the nanospray source, the spray voltage was set at 1.4 kV, and the capillary temperature was 275°C. All of the samples were measured in data-dependent acquisition mode. Each run was preceded by a blank MS run to monitor system background. The peptide masses were measured using a full scan survey (scan range of 300–1700 m/z, with 30 K FWHM resolution at m/z = 400, target AGC value of 1.00 × 10^6^ and maximum injection time of 500 ms). In parallel to the high-resolution full scan in Orbitrap, the data-dependent CID scans of the 10 most intense precursor ions were fragmented and measured in the linear ion trap (normalized collision energy of 35%, activation time of 10 ms, target AGC value of 1.00 × 10^4^, maximum injection time of 100 ms, isolation window of 2 Da). Parent masses obtained in the Orbitrap analyzer were automatically calibrated using a locked mass of 445.1200. The fragment ion masses were measured in the linear ion trap to obtain the maximum sensitivity and the maximum amount of MS/MS data. Dynamic exclusion was implemented with a repeat count of 1 and exclusion duration of 30 s. Raw files (triplicates) generated from mass spectrometry analysis were processed with Proteome Discoverer 1.4 (ThermoFisher Scientific). This software was used to search the data using an in-house Mascot server (version 2.4.1, Matrix Science Inc.) against the Mouse subset (16,696 sequences) of the SwissProt database (version 2014_11). Database searches were performed using the following settings: a maximum of two trypsin miscleavages allowed, methionine oxidation and N-terminal protein acetylation as variable modifications, and cysteine carbamido-methylation as a fixed modification. A peptide mass tolerance of 6 ppm and fragment mass tolerance of 0.8 Da were used for the search analysis. Only peptides with high stringency Mascot score threshold (identity, FDR < 1%) were used for protein identification. Only proteins that interact with full-length peptides and not with peptides lacking the last three amino acids or bead control are listed in S1 Table. Number of peptide-spectrum matches was indicated to show the relative amount of pulled down proteins.

### Yeast two-hybrid

Entry clones were used in a Gateway LR reaction to transfer the DNA coding for the full-length coding sequence or GRASP55Δ into the Y2H activation domain expression vector pACT2 [52]. DNA fragments encoding the cytoplasmic sequences of JAM-A, JAM-B (41 last residues) and JAM-C (48 residues) or lacking the sequence encoding the last three aa of the proteins (JAMsΔ constructs) were cloned into the Y2H binding domain expression vector pGBT9. The vectors were then co-transformed in the AH109 yeast strain (MATa, trp1-901, leu2-3, 112, ura3-52, his3-200, gal4Δ, gal80Δ, LYS2∷GAL1UAS-GAL1TATAHIS3, GAL2UAS-GAL2TATA-ADE2, URA3∷MEL1 UASMEL1TATA-lacZ, MEL1) using the lithium acetate method [53]. Following transformation, the yeast were plated onto synthetic complete (SC) medium lacking leucine (-L) and tryptophan (-W) and were incubated at 30°C for 4 to 5 days. The yeast clones were then transferred in liquid SC-WL for 3 days at 30°C with agitation to normalize the yeast cell concentration used for the phenotypic assay. The cells were then diluted 1/20 in water and spotted onto selective medium (-WHL) for the phenotypic assay.

### HTRF assay

The binding parameters for the JAM peptides to the fusion proteins were evaluated using the homogenous time-resolved fluorescence assay (HTRF). Peptide binding to the fusion proteins was measured in 0.05 M HEPES, 0.15 M NaCl, and 0.25% BSA (w/v) pH 7.3 at equilibrium (18 h, 4°C) in reaction mixtures consisting in: fusion proteins at the indicated concentrations (Fig 1D) or 2.5 x 10^−9^ M (Fig 6B), anti-GST or anti-6His antibody coupled to terbium cryptate (1 x 10^−9^ M), streptavidin-d2 (1.25 x 10^−9^ M) (Cisbio), and biotinylated peptide (6 x 10^−9^ M) with competing non-biotinylated peptide or organic compound at the indicated concentration. In the latter case, DMSO concentration was kept constant. Upon excitation of the reaction mixture at 337 nm, a 615 nm fluorescence emission is produced by the donor terbium that excites a 665 nm emission by the acceptor streptavidin-d2 bound to the biotinylated peptide, only if it resides in close vicinity to the donor, *i*.*e*. bound to the fusion protein. The intensity of light emission at 615 and 665 nm was measured using a Polarscan Omega (BMG Labtech) microplate reader equipped for HTRF. For each condition, the A665/A615 ratio (R) of fluorescence was calculated. The change in fluorescence, delta F (ΔF), was then computed as follows: [(R_Sample_-R_NSB_)/R_NSB_]x100, where R_NSB_ is the A665/A615 fluorescence ratio produced by the reaction mixture without fusion protein or biotinylated peptide. The EC_50_ was determined by plotting the ratio of the ΔF with homologous non-biotinylated or pharmacological inhibitor over ΔF_0_ (ΔF without competitor) against the log of the inhibitory compound using dose-response and curve-fitting analyses in Prism software (variable slope, four parameters). Values with an R square value greater than 0.99 were considered as significant.

### ITC assay

Isothermal titration calorimetry (ITC) was used to evaluate the thermodynamic parameters of the binding between GRASP55 and the selected JAM peptides. Purified GRASP55 was extensively dialyzed in 100 mM NaPO_4_ buffer at pH 7.5. Peptide powders were dissolved directly in the last protein dialysate prior to the experiments. The protein concentration was calculated by measuring the absorbance at 280 nm using a NanoDrop ND1000 (Thermo Scientific), and the titrations were conducted using a MicroCal ITC200 microcalorimeter (GE Healthcare). Each experiment was designed using a titrant concentration (peptide in the syringe) set at 10 to 30 times the analyte concentration (protein in the cell) and generally using 17 injections of 2.3 μL at 25°C (see S2 Table for details). A small initial injection (generally 0.2 μL) was included in the titration protocol to remove air bubbles trapped in the syringe prior to the titration. Integrated raw ITC data were fitted to a one-site nonlinear least-squares fit model using the MicroCal Origin plugin (http://www.originlab.com/) after subtraction of the control experiments (titration of the ligand from the syringe into the buffer) when necessary. Finally, the ΔG (G: Gibbs free energy) and TΔS (T: absolute temperature, S: entropy) values were calculated from the fitted ΔH (H: enthalpy) and K_A_ values using the following equations: ΔG = -R.T.lnK_A_ and ΔG = ΔH–TΔS.

### Protein crystallization

Purified GRASP55 was concentrated to approximately 15 mg/mL in a solution of 20 mM Tris^.^HCl pH 8.0, 150 mM NaCl for crystallization. Initial hits were obtained using commercially available sparse matrix screens (Hampton Research) using the sitting drop vapor diffusion method at 20°C. Optimization was conducted with the hanging drop vapor diffusion method, and diffraction-quality crystals were obtained in a solution of 2.0 M sodium formate, 0.1 M sodium acetate, pH 4.6. Crystals were soaked with JAM-B or JAM-C peptide using a 1:1 molar ratio for one day. The crystals were cryoprotected in reservoir solution supplemented with 25% glycerol and then flash frozen in liquid nitrogen. For structural characterization and refinement, see Supplemental Experimental Procedures.

### DSF assay

Differential scanning fluorimetry (DSF) was performed as previously described [54]. A protein/SYPRO orange dye mix containing 4 μM GRASP55 and a 1:5,000 dilution of dye (Life Technologies) were prepared in phosphate-buffered saline (PBS) extemporally. Then, 19.5 μL of the protein/dye mix was aliquoted into a 96-well plate, and 0.5 μL of Graspin (2 mM stock solution in 100% DMSO, 50 μM final concentration) or DMSO control (2.5% final DMSO concentration) was dispensed. The GRASP55/JAM-C DSF experiment was performed by adding JAM-C peptide (1 mM stock in PBS) to the protein/dye mix at a final concentration of 50 μM in the presence of 2.5% DMSO. After sealing with optical tape, thermal melting experiments were performed using a CFX96 (Bio-Rad) Real-time PCR detection system. The plates were first equilibrated at 25°C for 5 min and then heated at increments of 1°C every 60 s, from 20 to 90°C. The fluorescence intensity was recorded at every temperature step using the built-in FRET filter. Raw fluorescence data were evaluated using Microsoft Excel and GraphPad Prism template files adapted from Niesen *et al* [54]. After normalization, the melting temperatures (Tm) were measured using a Boltzmann fit equation in GraphPad Prism 5.03.

### Mice

Mice were used in compliance with the laws and protocols approved by the animal ethics committees (Agreement #02294.01). *Gorasp2*-deficient animals were generated as described in Supporting Information. Mice used in this study were backcrossed for more than six generation onto C57BL/6J background. Knock-out mice or littermate controls were obtained from heterozygous crossing.

### Sperm analysis

Sperm analysis was outsourced to Charles River Company. Sperm concentration, percentage of cells with normal morphology, abnormal head, bent midpiece, normal motility but also weight of testis, epididymis and seminal vesicle were determined.

### Graspin treatment

The GRASP55 inhibitor Graspin (PubChem CID #3113208, Vitas-M Laboratory Ltd., Ref. STK700118) was dissolved in 10% DMSO, 90% corn oil and was injected intraperitoneally into 27-day-old wild type male mice at 50 mg/kg on days 0, 3, 7, 10, and 14 (see Fig A in S8 Fig). Mice receiving Graspin or vehicle treatments were sacrificed 16 days after treatment initiation.

### Mouse embryonic fibroblast culture, transfection and treatment

*Gorasp2*^*+/+*^ and *Gorasp2*^*-/-*^ primary Mouse Embryonic Fibroblasts (MEFs) were isolated from 14-day embryos. The two uterine horns were collected in sterile conditions. Each embryo was released in PBS, head was collected for genotyping, liver and viscera were removed. Embryos were crushed on 70 μm cell strainer, washed in culture medium and seeded in 25 cm^2^ flask. MEFs were cultivated in DMEM supplemented with 10% fetal calf serum (FCS), 2 mM L-Glutamine, 100 U/ml penicillin-streptomycin, 1% essential amino acids, 25 μM β-mercaptoethanol and 1 mM sodium pyruvate at 37°C in a 5% CO_2_ humidified atmosphere. Re-expression of GRASP55 in *Gorasp2*^*-/-*^ MEFs was achieved upon transfection of GRASP FL fused to mCherry using MEF 2 Nucleofector Kit according to manufacturer instruction (Lonza). For Graspin treatment, MEFs plated at 70% confluency were incubated overnight and treatment was started the following morning for 48hours with Graspin at indicated concentrations. Graspin stock solution was dissolved at 10mM concentration in anhydrous DMSO (Cat# D12345, Life Technology). Treatment with DMSO 0.5% corresponding to the highest Graspin concentration (50μM) was used as control.

### Co-immunoprecipitation and immunoblotting

For co-immunoprecipitation of GRASP55 with JAM-C, *Gorasp2*^*+/+*^ and *Gorasp2*^*-/-*^ mouse testes were frozen in nitrogen, crushed with a pestle and solubilized in lysis buffer (50 mM HEPES pH 7.3, 10% glycerol, 0.1 mM EDTA, 150 mM NaCl, 1% Triton X100 and protease inhibitors). Protein G Sepharose 4 Fast Flow (GE Healthcare) was coupled to an anti-GRASP55 or rabbit IgG control antibody and incubated with pre-cleared testis lysate (5 mg/mL of proteins, approximately 45 mg per condition) overnight at 4°C. For GRASP55 immunoblotting, *Gorasp2*^*+/+*^ and *Gorasp2*^*-/-*^ mouse lung, heart and testis tissues were frozen in nitrogen, crushed with a pestle and solubilized in RIPA buffer (50 mM Tris HCL pH 7.5, 150 mM NaCl, 1% Triton X100, 0.1% SDS, 1% Na deoxycholate and protease inhibitors). Denatured proteins were separated by electrophoresis in 8% or 10% acrylamide gels and transferred to nitrocellulose membrane. The membranes were blocked with 5% non-fat dry milk, 0.05% Tween, and 1X PBS for 1 h at room temperature and incubated with primary antibodies overnight at 4°C, followed by secondary antibodies for 1 h at room temperature (RT).

### Immuno-histochemistry and Periodic Acid Schiff staining

Testes were carefully collected, and surrounding tissues were removed. The organs were fixed in 4% paraformaldehyde in PBS overnight and conserved in ethanol 70% before paraffin-embedding. 3-μm-thick deparaffinized sections were stained with PAS or used for immunofluorescence. Primary antibodies were incubated overnight at 4°C, and secondary antibodies were incubated for 1 h RT. Same-species primary antibodies (pAb 501 rabbit anti-mouse JAM-C and rabbit anti-mouse GRASP55 antibodies in Figs 3 and 7) were detected using tyramide signal amplification (TSA) according to manufacturer instructions (PerkinElmer Inc.). The slides were mounted with Prolong Gold Antifade Reagent (Invitrogen). For JAM-A, ZO-1 and SOX 9 staining, testes were fixed in 4% paraformaldehyde in PBS overnight, washed in PBS and transferred in 30% sucrose overnight before soaking, embedding and freezing in gelatin-sucrose solution (7.5% and 15% respectively, in PBS). Sections (14-μm-thick) were generated with a CryoStar NX70 cryostat (Thermo Scientific). IF were performed in same conditions as previously described. Detection of apoptotic cells on testis section after Graspin treatment was assessed by TUNEL staining according to manufacturer instructions (DeadEnd Fluorometric TUNEL System, Promega). Images were acquired using LSM510 META and LSM880 AiryScan confocal microscopes (Zeiss) and analyzed using Zen, ImageJ and Adobe Photoshop software. Periodic Acid Schiff coloration of testis sections was performed on Bouin’s solution fixed tissues as previously described [55] using hematoxylin-eosin as counterstain.

### Immunostaining on isolated sperm cells

Intact epididymes (caput, corpus and cauda) were collected from *Gorasp2*^*+/+*^, *Gorasp2*^*-/-*^, vehicle or Graspin treated mice. Epididymes were minced and placed into 500μl of PBS at 37°C from 30 min to allow sperm to swim-out. Diffused cell suspension were filtered through a 70μM cell stainer and resuspended in 500μl PBS solution. 50 μL of cell suspension were loaded into a cytospin chamber and centrifuged for 10 min at 250 rpm on poly L-Lysine coated slides. After centrifugation, supernatant were removed and cells on microscope glass slides were fixed 10 min in ice-cold methanol. Then, cells were washed in PBS and incubated with primary antibody solution (JAM-C) overnight at 4°C and secondary antibody, PNA-FITC and DAPI solution 1h, RT. The slides were mounted with Prolong Gold Antifade Reagent (Invitrogen).

### Germ cell quantification by flow cytometry

Seminiferous tubules from decapsulated testes or epididymes were minced in PBS, warmed to 37°C and incubated for 15 min at room temperature with agitation (200 rpm on an orbitary shaker). Diffused germ cell suspension were collected in PBS, filtered through a 70μM cell strainer (Ref 352350, BD Falcon), fixed and permeabilized using the Cytofix/Cytoperm kit (BD Biosciences). DNA was stained by incubation with DAPI for 30 min at room temperature. Flow cytometry analysis was performed using a BD-FORTESSA (BD Biosciences) cytometer, and the results were analyzed using BD-DIVA version 8 (BD Bioscience), FlowJo version 10 (TreeStar) and Kaluza version 1.3 (Beckman Coulter Inc.) softwares.

### Statistical analysis

Data were analyzed for statistical significance using GraphPad Prism software with the methods that are mentioned in figure legends.

## Supporting information

S1 Table Table showing the list of proteins identified by peptide pulldown and mass spectrometry. (DOCX)

S2 Table Table comparing the affinity measurements by ITC. (DOCX)

S3 Table Crystallographic data collection and refinement statistics for the two PDB structures, bound to JAM-B and JAM-C. (DOCX)

S1 Fig Generation and characterization of *Gorasp2*^-/-^ mice. **(A)** The strategy used to generate *Gorasp2* knock-out mice is shown. The endogenous *Gorasp2* locus contains 13 exons (only 6 represented—black boxes) and introns (black lines). The targeting vector contained a diphtheria toxin A-negative selection cassette (DTA) followed by *Gorasp2* sequences (exon 3 to exon 6) flanked by LoxP recombination sites (green) and a neomycin-positive selection cassette (NeoR) flanked by FLP recombination target (FRT) sites (blue). The recombined *Gorasp2* locus was obtained by homologous recombination. F1 breeding with CMV-Cre deleter mice resulted in knock-out of the *Gorasp2* locus by Cre recombination (dashed black lines). The hybridization sites, PCR primer names and amplified fragment sizes are indicated in red. **(B)** Agarose gel of the PCR-amplified products used for *Gorasp2* strain genotyping. Results obtained for homozygous (-/-), wild-type (+/+) and heterozygous (+/-) mice are shown. **(C)** Immunoblot of GRASP55 using total tissue lysates from the indicated organs isolated from *Gorasp2*^*+/+*^ and *Gorasp2*^*-/-*^ mice. Actin is shown as loading control. **(D)** Growth curves of *Gorasp2*^*+/+*^ (solid line) and *Gorasp2*^*-/-*^ (dashed line) mice, illustrating growth retardation in *Gorasp2*^*-/-*^ mice. Each point represents the mean weight ± s.e.m. of 4 mice. Mann-Whitney test; *: *P*<0.05. **(E)** Testis weight of *Gorasp2*^*+/+*^ and *Gorasp2*^*-/-*^ adult mice (12 weeks old, n = 16 and n = 18 respectively) expressed as percentage of body weight. **(F-G)** Epididymis weight **(F)** and seminal vesicle weight **(G)** from *Gorasp2*^*+/+*^ and *Gorasp2*^*-/-*^ adult mouse testes (n = 6 and n = 4, respectively). **(H)** Sperm concentration from *Gorasp2*^*+/+*^, *Gorasp2*^*+/-*^ and *Gorasp2*^*-/-*^ of adult (12 weeks old) mouse testes (n = 6, n = 6 and n = 4, respectively). **(I-K)** Properties of sperm cells isolated from epididymis of *Gorasp2*^*+/+*^ and *Gorasp2*^*-/-*^ mice. Percentage of cells with bent midpiece **(I)** or abnormal head **(J)** and percentage of motile cells **(K)** are shown. (n = 6 and n = 4, respectively). Student’s unpaired *t*-test; *: *P*<0.05, ***: *P*<0.001.
(TIF)

S2 Fig Periodic Acid Schiff—Hematoxylin-Eosin (PAS-HE) staining of *Gorasp2*^*+/+*^ and *Gorasp2*^*-/-*^ adult mouse testes sections. Representative pictures of seminiferous tubules at stage III, VII, VIII and X are shown. Seminiferous tubule staging was performed according to morphological criteria as described in Meistrich and Hess [56]. In inserts, arrowheads show acrosomal staining of developing wild type spermatids. Scale bars: main panels, 20 μm; inserts, 10 μm.
(TIF)

S3 Fig JAM-C and JAM-B expression in tubule sections from *Gorasp2*^*-/-*^ and *Gorasp2*^*+/+*^ mice. **(A)** Confocal images of JAM-C and DAPI staining of seminiferous tubule sections of adult *Gorasp2*^*+/+*^ and *Gorasp2*^*-/-*^ mice. White arrowheads indicate polarized JAM-C expression in developing round spermatids, red arrowheads indicate JAM-C expression in spermatogonia and primary spermatocytes. Seminiferous tubule stages are indicated. Scale bar, 20 μm. **(B)** Confocal images of JAM-B (green), JAM-C (red) and DAPI (blue) staining of seminiferous tubule sections of adult *Gorasp2*^*+/+*^ and *Gorasp2*^*-/-*^ mice. Scale bars: main panels, 50 μm; high magnification, 5μm.
(TIF)

S4 Fig Nectin3, JAM-A, ZO-1 and SOX9 stainings of tubule sections from *Gorasp2*^*-/-*^ and *Gorasp2*^*+/+*^ mice. **(A)** Confocal images of Nectin3, PNA and DAPI staining of seminiferous tubule sections of adult *Gorasp2*^*+/+*^ and *Gorasp2*^*-/-*^ mice. Note the lack of apical ectoplasmic specialization stained with Nectin3 in *Gorasp2* deficient mice. Scale bars: main panels, 50 μm; high magnification, 5μm. **(B)** GRASP55, JAM-A and DAPI staining of seminiferous tubule sections of adult *Gorasp2*^*+/+*^ and *Gorasp2*^*-/-*^ mice. Scale bar, 100 μm. **(C)** Confocal images of ZO-1, JAM-A and DAPI staining of seminiferous tubule sections of adult *Gorasp2*^*+/+*^ and *Gorasp2*^*-/-*^ mice. Scale bar, 20 μm. **(D)** Confocal images of SOX 9 staining of seminiferous tubule sections of adult *Gorasp2*^*+/+*^ and *Gorasp2*^*-/-*^ mice. Numbers of stained nucleus per tubules ± s.e.m. are indicated. Counting was done on a single large mosaic picture (1080x1080μm) obtained on testes sections obtained from mice with the indicated genotype. Scale bar, 100 μm.
(TIF)

S5 Fig Image analysis workflow used for *in vitro* Golgi morphology studies. **(A)** Immunofluorescence visualization of GRASP55 and GM130 in wild type *Gorasp2*^*+/+*^ and homozygous *Gorasp2*^*-/-*^ MEFs transfected or not with mCherry GRASP55. DNA is labelled with DAPI. Scale bars, 5 μm. **(B)** Golgi density quantification in MEFs. Analysis was done using homemade automated Matlab script to treat batches of pictures as follow (available upon request). Color channels are first splitted (Step 1), smoothened by median filtering (Step 2) and binarized with automatic threshold for Golgi or with threshold equal to min intensity for nucleus (Step 3). Holes are then filled to quantify respective total areas of Golgi and nucleus (Step 4). Golgi density is expressed as ratio of Golgi stacks area (Step 3, upper panel) divided by total area of the Golgi (Step 4, upper panel). Cell to cell variations are normalized to the nucleus area of individual cells (Step 4, lower panel). Around 20 to 100 Golgi are analyzed per condition for each experiment. **(C)** Quantification of Golgi density in *Gorasp2*^*+/+*^ and *Gorasp2*^*-/-*^ MEFs transfected or not with mCherry GRASP55. Each circle represents one Golgi. Data are the mean ± s.e.m. of pooled results of three independent experiments (analysis of 20–100 Golgi per condition and experiment). Student’s unpaired *t*-test; n.s.: *P*>0.05, *: *P*<0.05, ***: *P*<0.001. **(D)** Examples of Golgi density scores associated with images of Golgi stained with GM130 in MEFs.
(TIF)

S6 Fig Structural properties of the GRASP55/JAM interaction. **(A)** The PDZ1 domains were superimposed, and the amplitude of the PDZ2 movement between the ‘open’ (salmon) and ‘closed’ (blue) conformations was computed using the HINGEFIND algorithm (http://biomachina.org/disseminate/hingefind/hingefind.html) and in-house VMD plugins. The results revealed a root mean square deviation of 12.1 Å and a rotation of approximately 33 degrees. The three lowest frequency normal modes were computed for the ‘open’ form using elastic network models (http://www.sciences.univ-nantes.fr/elnemo/). The third normal mode provided the major contribution to the transition between the two states. **(B)** Detailed view of the 2Fo-Fc electron density map contoured at 1σ around JAM-B (blue) and its surrounding GRASP55 PDZ1 (yellow) and PDZ2 (salmon) residues. **(C)** Detailed view of the 2Fo-Fc electron density map for the GRASP55 PDZ12/JAM-C complex. **(D)** LigPlot+ representation of the complex highlighting the hydrogen bonding interaction contacts (green) and the van der Waals and hydrophobic contacts (semi circles). Contact residues highlighted in red are shared in the JAM-B and JAM-C complex structures (see Fig 5C for comparison).
(TIF)

S7 Fig Graspin treatment. **(A)** Schematic representation of the protocol used for Graspin treatment. Graspin, dissolved in 10% DMSO, 90% corn oil, was injected in 27-day old C57BL/6J mice at 50 mg/kg on days 0, 3, 7, 10, and 14. Animals were sacrificed on day 16. Blood samples were collected the day before the first injection (day -1) and at endpoint (day 16). **(B)** Toxicity in Graspin-treated mice was evaluated by weight loss and hematological toxicity. No significant differences in weight, white blood cell count (WBC) or hematocrit were observed after two weeks of Graspin treatment. **(C)** Seminiferous tubule quantification at each stage from confocal pictures of mice treated with vehicle or Graspin. Staging was based on PNA staining as described in Nakata et al. **(D)** Representative confocal images of peanut agglutinin (PNA) and DAPI staining of seminiferous tubule sections used for quantification in (C). Seminiferous tubule stages are indicated. Scale bar, 100 μm. **(E)** Quantification of elongated spermatids (ES) per tubule (at stage V to VIII) of mice treated with vehicle or Graspin. Student’s unpaired *t*-test; ***: *P*<0.001. **(F)** Flow cytometry quantification of germ cell numbers isolated from testes of 27-days old mice treated with Graspin for two weeks and analyzed two days after the last Graspin injection. Vehicle, n = 10; Graspin, n = 10. Student’s unpaired *t*-test; **: *P*<0.01, ***: *P*<0.001.
(TIF)

S8 Fig Graspin treatment does not induce apoptosis. Representative large mosaic pictures (735 x 735μm) of TUNEL staining (green) obtained on testes sections from vehicle or Graspin treated mice. Rare cells are stained by TUNEL, indicating that Graspin does not induce apoptosis. Scale bar, 100 μm.
(TIF)

S1 Text Supplementary methods and references. (DOCX)
