# Supplementary material for: Genetic, structural, and chemical insights into the dual function of GRASP55 in germ cell Golgi remodeling and JAM-C polarized localization during spermatogenesis
Source: PLoS Genet. 2017 Jun 15;13(6):e1006803. doi: 10.1371/journal.pgen.1006803 (PMC5472279; doi:10.1371/journal.pgen.1006803)
Supplement: S1 Table — (DOCX) [file pgen.1006803.s001.docx]

| Protein | SwissProt  number | PDZ  domain | Number of peptide-spectrum matches | | | | | |
| --- | --- | --- | --- | --- | --- | --- | --- | --- |
|  |  |  | JAM-A | JAM-AΔ | JAM-B | JAM-BΔ | JAM-C | JAM-CΔ |
| ZO-2 | Q9Z0U1 | yes | 261 | - | 198 | - | 247 | - |
| ZO-1 | P39447 | yes | 142 | - | 148 | - | 154 | - |
| GRASP55 | Q99JX3 | yes | 93 | - | 120 | 3 | 110 | - |
| RADIL | Q69Z89 | yes | 18 | - | - | - | - | - |
| Syntenin1 | O08992 | yes | 17 | - | - | - | - | - |
| GRIP1 | Q925T6 | yes | 10 | - | 19 | - | 43 | - |
| LNX2 | Q91XL2 | yes | 10 | - | - | - | - | - |
| HTRA1 | Q9R118 | yes | 6 | - | - | - | - | - |
| PTN13 | Q64512 | yes | 4 | - | - | - | - | - |
| PICK1 | Q62083 | yes | 2 | - | - | - | - | - |
| BSPRY | Q80YW5 | no | - | - | 121 | - | 165 | - |

The list is restricted to proteins that were differentially identified with native (JAMs) and mutant peptides devoid of PDZ-binding motifs (JAMsΔ).
