# Supplementary material for: Genetic, structural, and chemical insights into the dual function of GRASP55 in germ cell Golgi remodeling and JAM-C polarized localization during spermatogenesis
Source: PLoS Genet. 2017 Jun 15;13(6):e1006803. doi: 10.1371/journal.pgen.1006803 (PMC5472279; doi:10.1371/journal.pgen.1006803)
Supplement: S2 Table — (DOCX) [file pgen.1006803.s002.docx]

| Name | Concentrations (µM) | | T  (K) | n | ΔG (Kcal.mol^-1^) | ΔH (Kcal.mol^-1^) | -TdS (Kcal.mol^-1^) | Kd  (µM) |
| --- | --- | --- | --- | --- | --- | --- | --- | --- |
|  | Cell | Syringe |  |  |  |  |  |  |
| GST-GRASP55 FL vs JAM-A | 100 | 1000 | 298 | 0.8 | -6.3 ± 0.1 | -6.7 ± 0.3 | 0.5 ± 0.3 | 27.0 ± 3.0 |
| GST-GRASP55 FL vs JAM-B | 70 | 800 | 298 | 0.9 | -7.2 ± 0.1 | -6.1 ± 0.1 | -1.2 ± 0.1 | 4.9 ± 0.3 |
| GST-GRASP55 FL vs JAM-C | 70 | 800 | 298 | 1.0 | -7.4 ± 0.1 | -5.3 ± 0.1 | -2.1 ± 0.1 | 3.7 ± 0.3 |
| His-GRASP55 PDZ12 vs JAM-A | 150 | 2250 | 298 | 0.9 | -6.6 ± 0.1 | -5.7 ± 0.1 | -0.9 ± 0.1 | 14.0 ± 1.0 |
| His-GRASP55 PDZ12 vs JAM-B | 150 | 1500 | 298 | 1.3 | -8.1 ± 0.1 | -8.1 ± 0.1 | 0.1 ± 0.1 | 1.2 ± 0.1 |
| His-GRASP55 PDZ12 vs JAM-C | 100 | 1000 | 298 | 1.0 | -7.0 ± 0.1 | -8.4 ± 0.1 | 1.4 ± 0.1 | 7.1 ± 0.1 |
| GRASP55 PDZ12 vs JAM-C | 100 | 1000 | 298 | 1.1 | -7.4 ± 0.1 | -6.5 ± 0.1 | -0.9 ± 0.1 | 3.8 ± 0.1 |

Using of JAM-A, JAM-B and JAM-C with recombinant GST-tagged GRASP55 full-length (GST-GRASP55 FL), Histidine-tagged PDZ tandem of GRASP55 (His-GRASP55 PDZ12), or untagged PDZ tandem of GRASP55.
