# Supplementary material for: Genetic, structural, and chemical insights into the dual function of GRASP55 in germ cell Golgi remodeling and JAM-C polarized localization during spermatogenesis
Source: PLoS Genet. 2017 Jun 15;13(6):e1006803. doi: 10.1371/journal.pgen.1006803 (PMC5472279; doi:10.1371/journal.pgen.1006803)
Supplement: S3 Table — (DOCX) [file pgen.1006803.s003.docx]

|  | GRASP55-HIS / JAM-B | GRASP55-HIS / JAM-C |
| --- | --- | --- |
| Data collection  Space group  Cell dimensions *a*,*b*,*c* (Å)  Resolution (Å)  *R*_sym_  *I/σI*  Completeness (%)  Redundancy | I4_1_22  133.0, 133.0, 219.4  54.85-2.986 (3.093-2.986)  0.179 (0.775)  11.66 (4.14)  99.93 (99.85)  13.6 (14.1) | I4_1_22  132.5, 132.5, 220.7  71.42-2.71 (2.81-2.71)  0.137 (0.667)  7.10 (2.38)  99.85 (100)  6.1 (6.3) |
| Refinement  Resolution (Å)  *R*_work_ / *R*_free_ | 54.85-2.986  22.47 / 27.39 | 71.42-2.71  25.64 / 29.14 |
| No. atoms  Protein  Water | 3610  3577  33 | 3614  3525  89 |
| B-factors  Protein  Ligand / ion  Water | 69.7  69.9  53.3 | 72.6  73.0  56.7 |
| R.m.s deviations  Bond lengths (Å)  Bond angles (°) | 0.005  1.17 | 0.005  1.07 |
| Deposition  PDB ID | 5GMJ | 5GMI |

The highest resolution shell is shown in brackets.
