# Supplementary figures and images for: Genetic, structural, and chemical insights into the dual function of GRASP55 in germ cell Golgi remodeling and JAM-C polarized localization during spermatogenesis
Source: PLoS Genet. 2017 Jun 15;13(6):e1006803. doi: 10.1371/journal.pgen.1006803 (PMC5472279; doi:10.1371/journal.pgen.1006803)

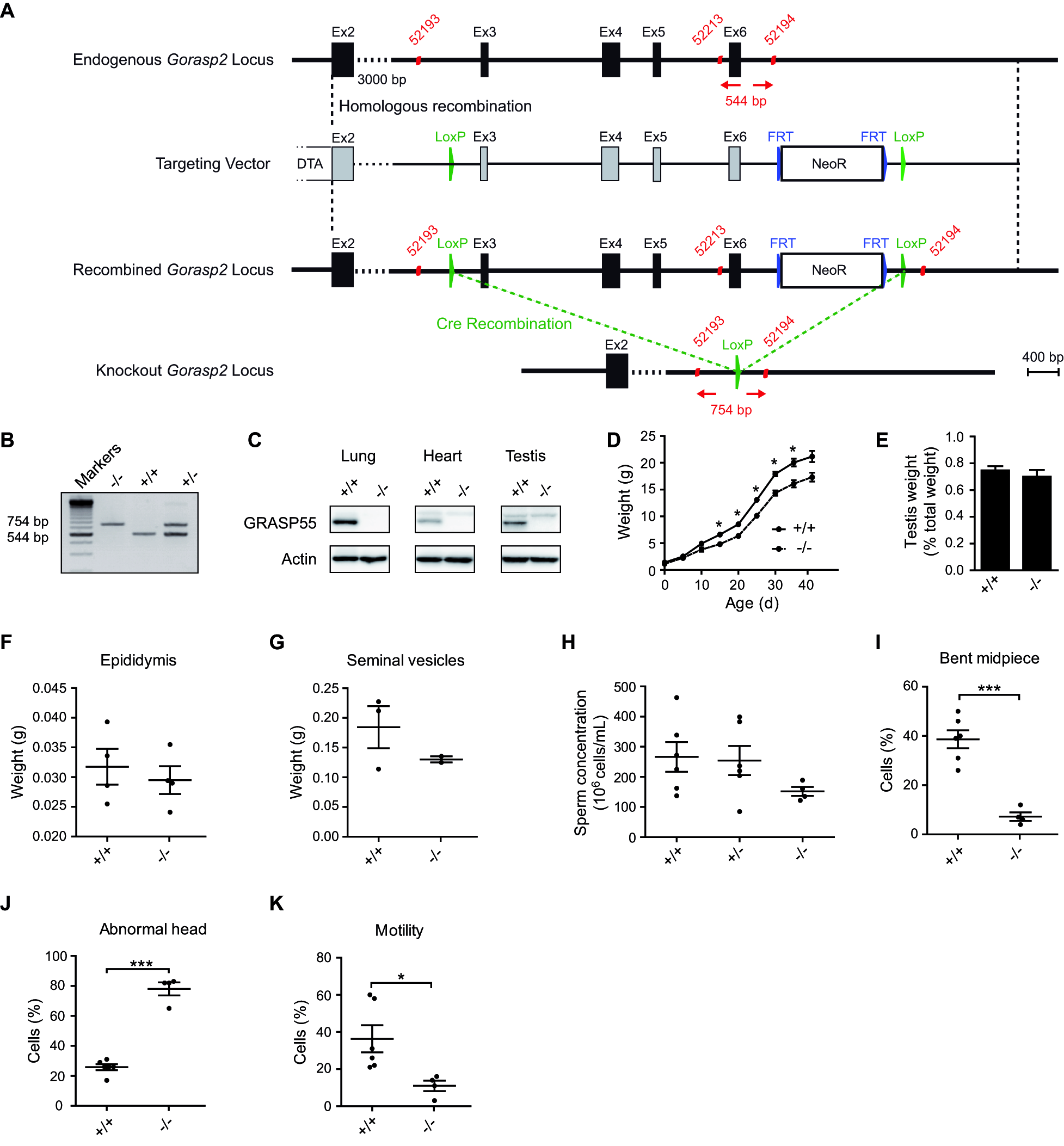

Supplement: S1 Fig — (A) The strategy used to generate Gorasp2 knock-out mice is shown. The endogenous Gorasp2 locus contains 13 exons (only 6 represented—black boxes) and introns (black lines). The targeting vector contained a diphtheria toxin A-negative selection cassette (DTA) followed by Gorasp2 sequences (exon 3 to exon 6) flanked by LoxP recombination sites (green) and a neomycin-positive selection cassette (NeoR) flanked by FLP recombination target (FRT) sites (blue). The recombined Gorasp2 locus was obtained by homologous recombination. F1 breeding with CMV-Cre deleter mice resulted in knock-out of the Gorasp2 locus by Cre recombination (dashed black lines). The hybridization sites, PCR primer names and amplified fragment sizes are indicated in red. (B) Agarose gel of the PCR-amplified products used for Gorasp2 strain genotyping. Results obtained for homozygous (-/-), wild-type (+/+) and heterozygous (+/-) mice are shown. (C) Immunoblot of GRASP55 using total tissue lysates from the indicated organs isolated from Gorasp2+/+ and Gorasp2-/- mice. Actin is shown as loading control. (D) Growth curves of Gorasp2+/+ (solid line) and Gorasp2-/- (dashed line) mice, illustrating growth retardation in Gorasp2-/- mice. Each point represents the mean weight ± s.e.m. of 4 mice. Mann-Whitney test; *: P<0.05. (E) Testis weight of Gorasp2+/+ and Gorasp2-/- adult mice (12 weeks old, n = 16 and n = 18 respectively) expressed as percentage of body weight. (F-G) Epididymis weight (F) and seminal vesicle weight (G) from Gorasp2+/+ and Gorasp2-/- adult mouse testes (n = 6 and n = 4, respectively). (H) Sperm concentration from Gorasp2+/+, Gorasp2+/- and Gorasp2-/- of adult (12 weeks old) mouse testes (n = 6, n = 6 and n = 4, respectively). (I-K) Properties of sperm cells isolated from epididymis of Gorasp2+/+ and Gorasp2-/- mice. Percentage of cells with bent midpiece (I) or abnormal head (J) and percentage of motile cells (K) are shown. (n = 6 and n = 4, respectively). Student’s unpaired t-test; *: P [file pgen.1006803.s004.tif]

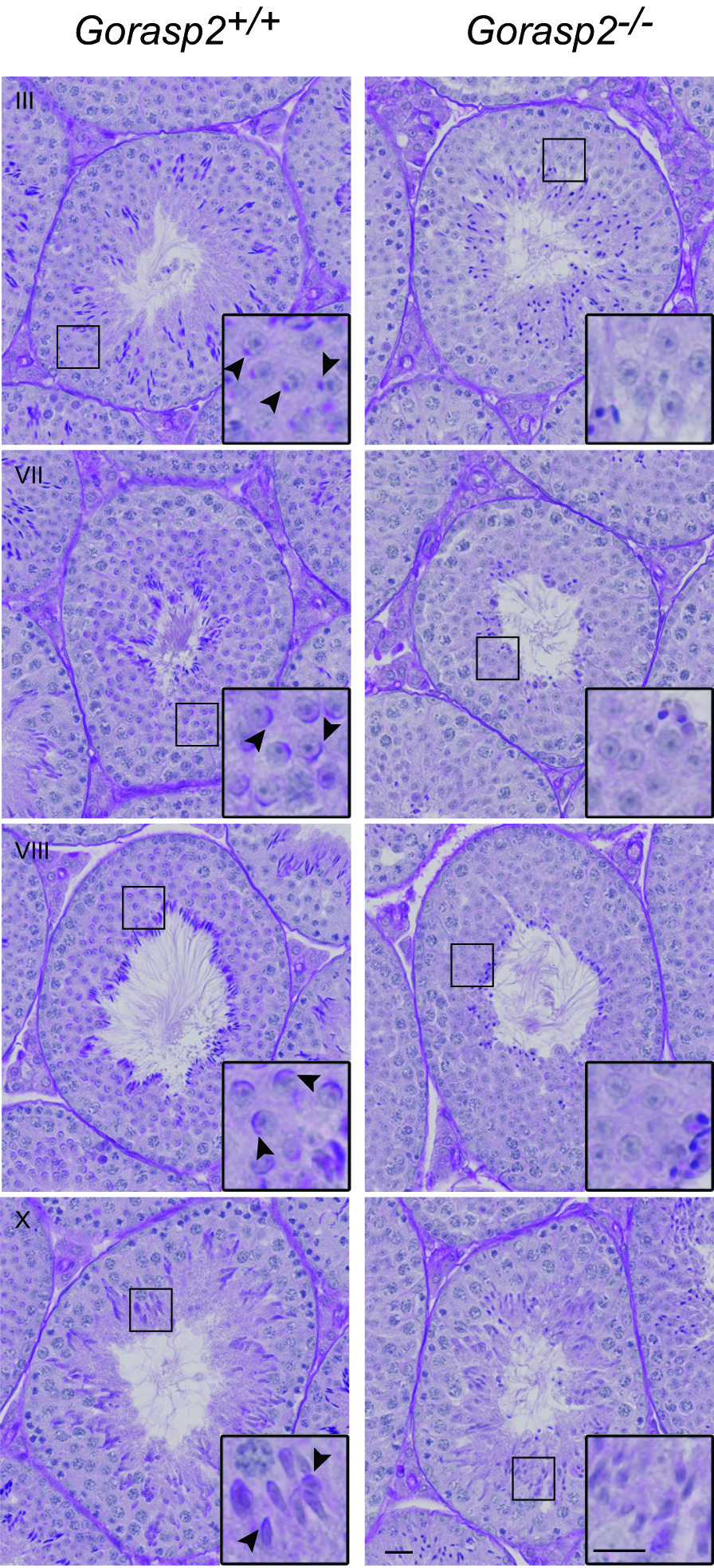

Supplement: S2 Fig — Representative pictures of seminiferous tubules at stage III, VII, VIII and X are shown. Seminiferous tubule staging was performed according to morphological criteria as described in Meistrich and Hess [56]. In inserts, arrowheads show acrosomal staining of developing wild type spermatids. Scale bars: main panels, 20 μm; inserts, 10 μm. (TIF) [file pgen.1006803.s005.tif]

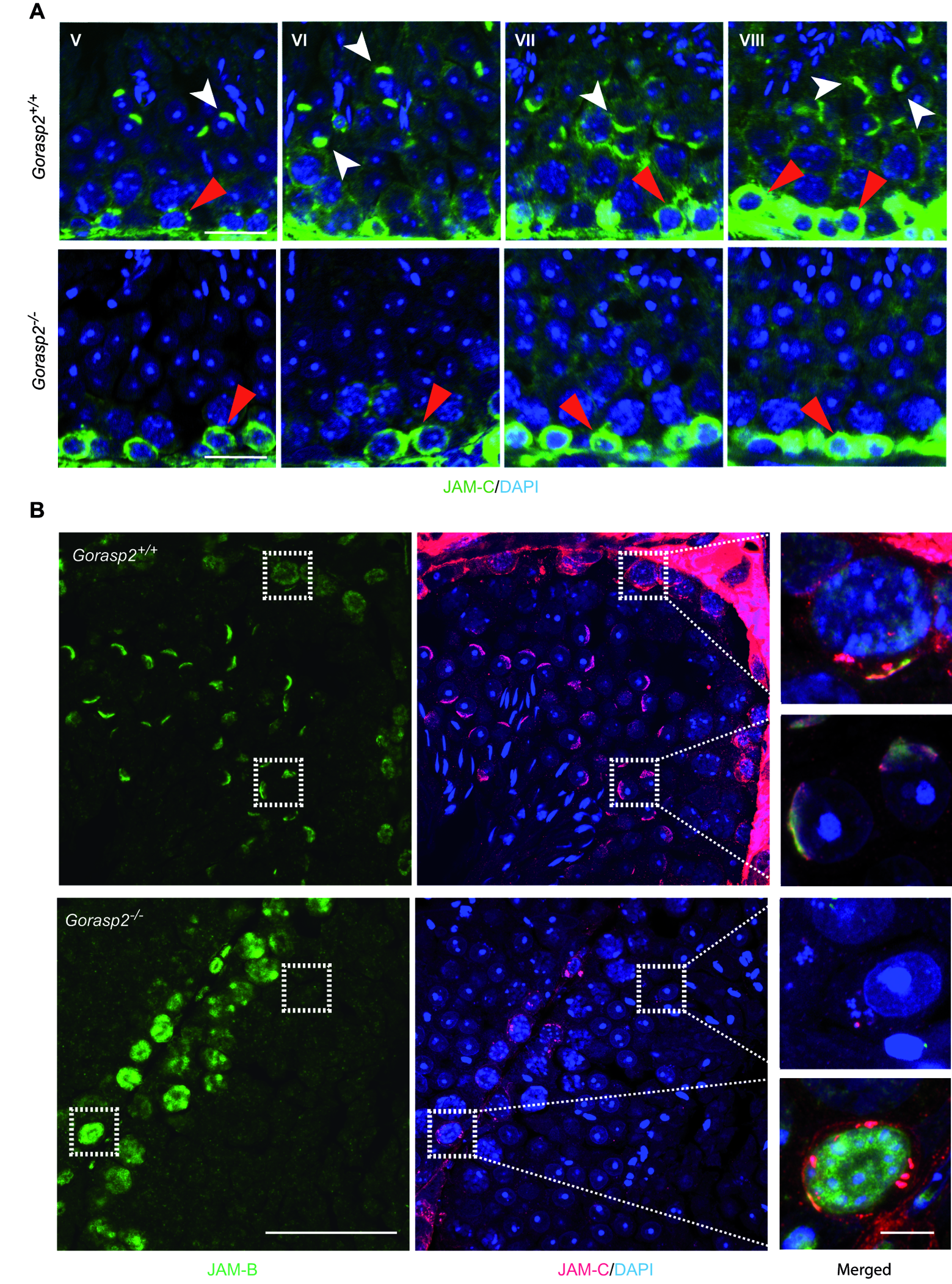

Supplement: S3 Fig — (A) Confocal images of JAM-C and DAPI staining of seminiferous tubule sections of adult Gorasp2+/+ and Gorasp2-/- mice. White arrowheads indicate polarized JAM-C expression in developing round spermatids, red arrowheads indicate JAM-C expression in spermatogonia and primary spermatocytes. Seminiferous tubule stages are indicated. Scale bar, 20 μm. (B) Confocal images of JAM-B (green), JAM-C (red) and DAPI (blue) staining of seminiferous tubule sections of adult Gorasp2+/+ and Gorasp2-/- mice. Scale bars: main panels, 50 μm; high magnification, 5μm. (TIF) [file pgen.1006803.s006.tif]

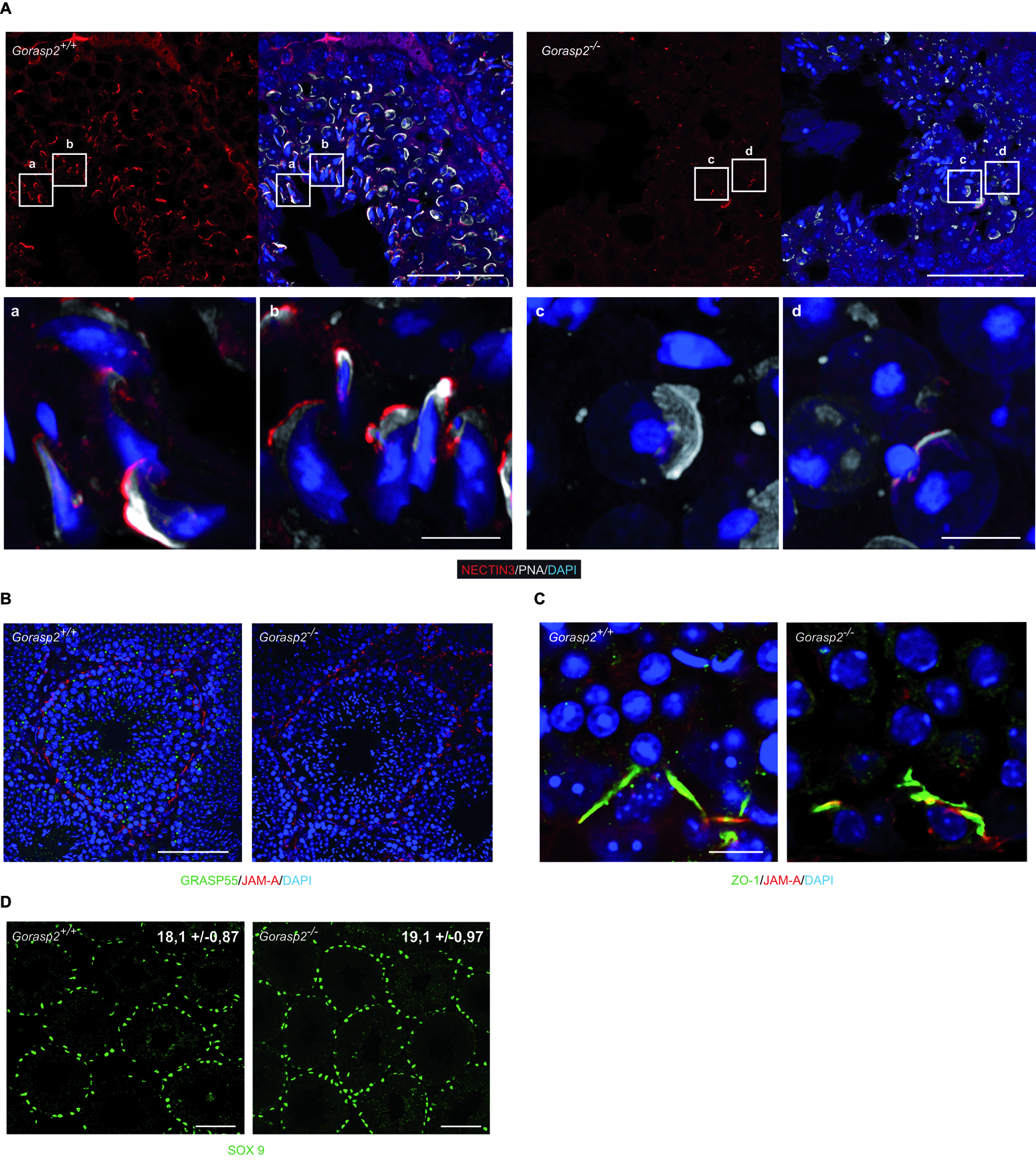

Supplement: S4 Fig — (A) Confocal images of Nectin3, PNA and DAPI staining of seminiferous tubule sections of adult Gorasp2+/+ and Gorasp2-/- mice. Note the lack of apical ectoplasmic specialization stained with Nectin3 in Gorasp2 deficient mice. Scale bars: main panels, 50 μm; high magnification, 5μm. (B) GRASP55, JAM-A and DAPI staining of seminiferous tubule sections of adult Gorasp2+/+ and Gorasp2-/- mice. Scale bar, 100 μm. (C) Confocal images of ZO-1, JAM-A and DAPI staining of seminiferous tubule sections of adult Gorasp2+/+ and Gorasp2-/- mice. Scale bar, 20 μm. (D) Confocal images of SOX 9 staining of seminiferous tubule sections of adult Gorasp2+/+ and Gorasp2-/- mice. Numbers of stained nucleus per tubules ± s.e.m. are indicated. Counting was done on a single large mosaic picture (1080x1080μm) obtained on testes sections obtained from mice with the indicated genotype. Scale bar, 100 μm. (TIF) [file pgen.1006803.s007.tif]

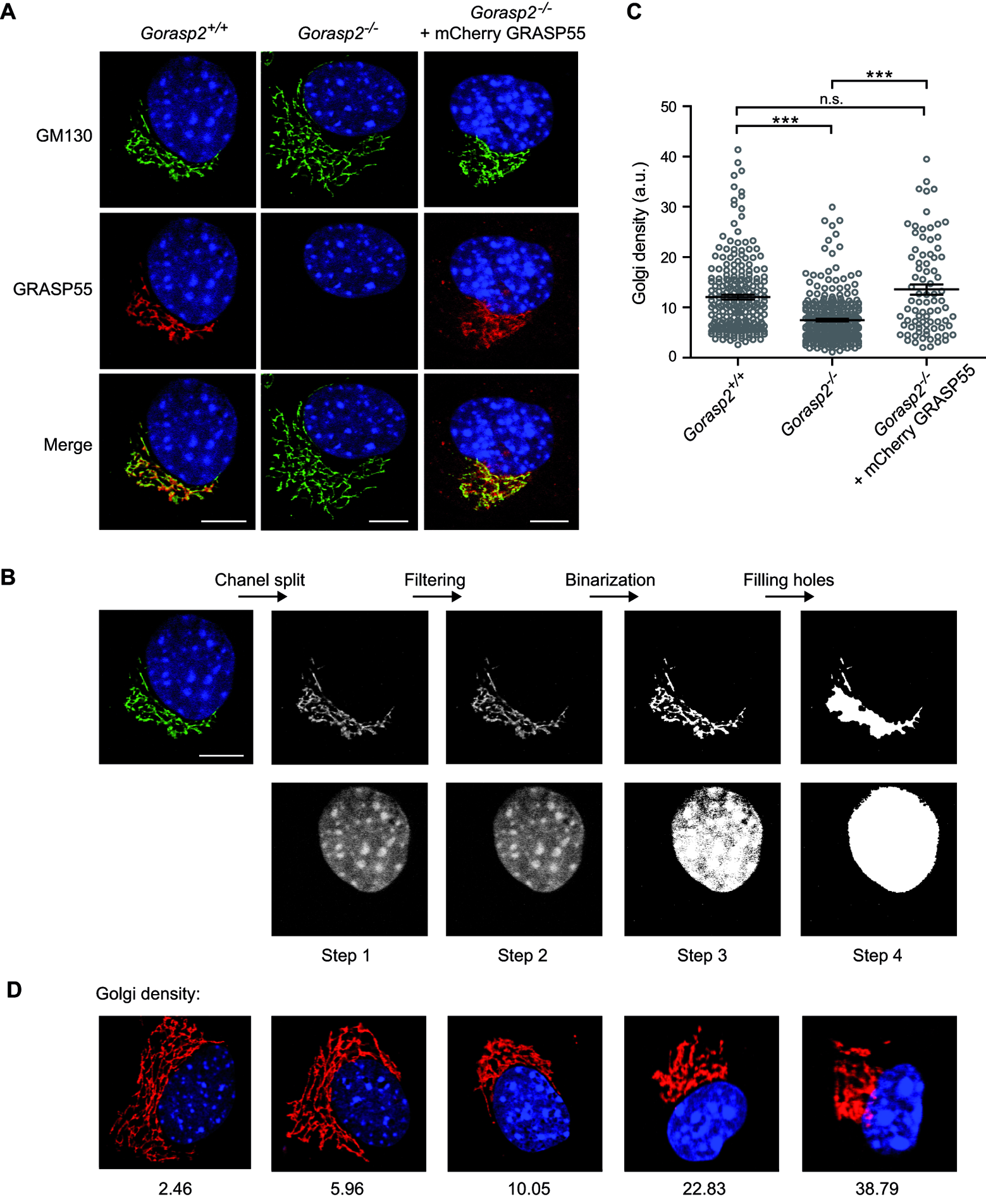

Supplement: S5 Fig — (A) Immunofluorescence visualization of GRASP55 and GM130 in wild type Gorasp2+/+ and homozygous Gorasp2-/- MEFs transfected or not with mCherry GRASP55. DNA is labelled with DAPI. Scale bars, 5 μm. (B) Golgi density quantification in MEFs. Analysis was done using homemade automated Matlab script to treat batches of pictures as follow (available upon request). Color channels are first splitted (Step 1), smoothened by median filtering (Step 2) and binarized with automatic threshold for Golgi or with threshold equal to min intensity for nucleus (Step 3). Holes are then filled to quantify respective total areas of Golgi and nucleus (Step 4). Golgi density is expressed as ratio of Golgi stacks area (Step 3, upper panel) divided by total area of the Golgi (Step 4, upper panel). Cell to cell variations are normalized to the nucleus area of individual cells (Step 4, lower panel). Around 20 to 100 Golgi are analyzed per condition for each experiment. (C) Quantification of Golgi density in Gorasp2+/+ and Gorasp2-/- MEFs transfected or not with mCherry GRASP55. Each circle represents one Golgi. Data are the mean ± s.e.m. of pooled results of three independent experiments (analysis of 20–100 Golgi per condition and experiment). Student’s unpaired t-test; n.s.: P>0.05, *: P<0.05, ***: P<0.001. (D) Examples of Golgi density scores associated with images of Golgi stained with GM130 in MEFs. (TIF) [file pgen.1006803.s008.tif]

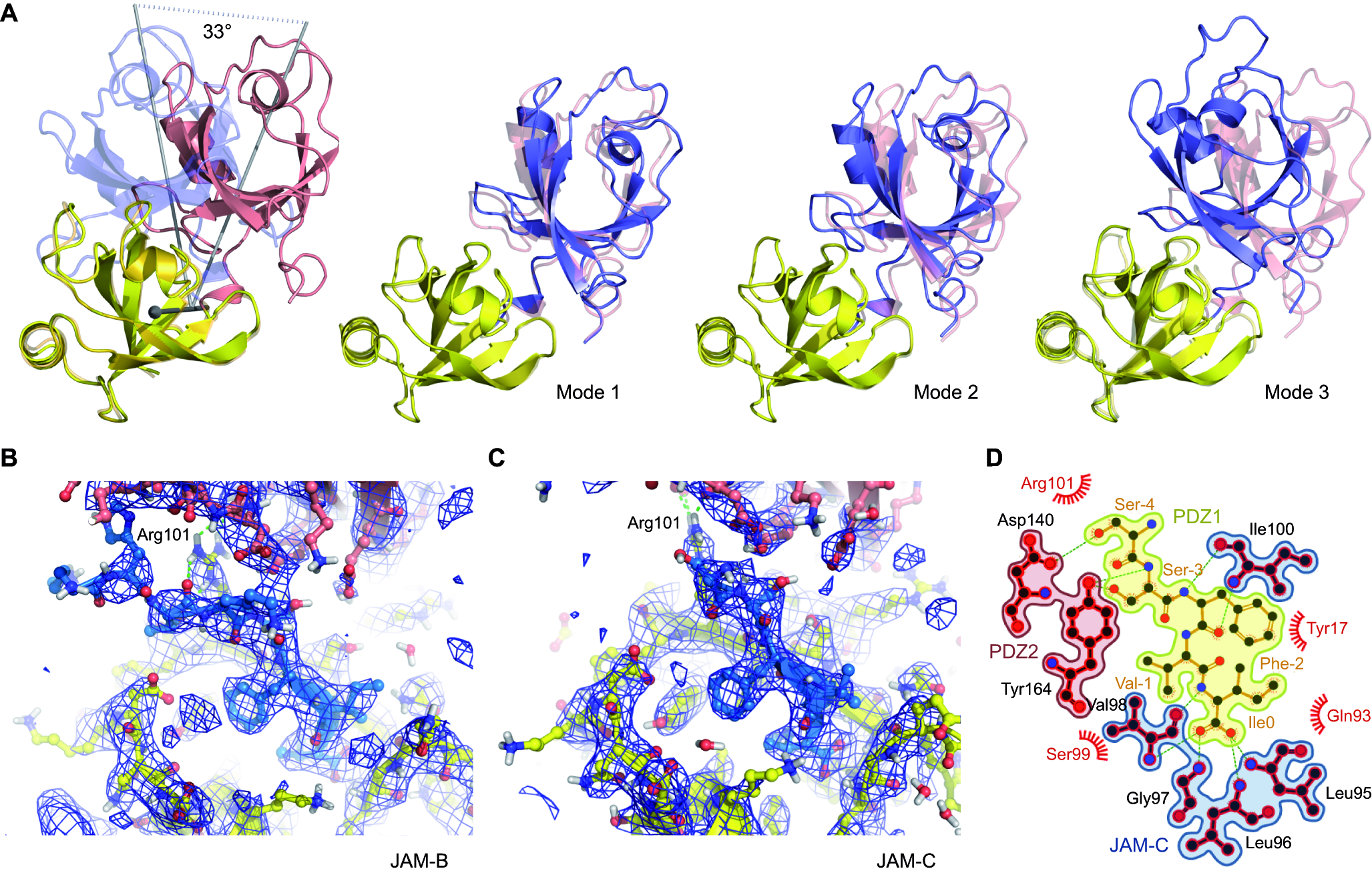

Supplement: S6 Fig — (A) The PDZ1 domains were superimposed, and the amplitude of the PDZ2 movement between the ‘open’ (salmon) and ‘closed’ (blue) conformations was computed using the HINGEFIND algorithm (http://biomachina.org/disseminate/hingefind/hingefind.html) and in-house VMD plugins. The results revealed a root mean square deviation of 12.1 Å and a rotation of approximately 33 degrees. The three lowest frequency normal modes were computed for the ‘open’ form using elastic network models (http://www.sciences.univ-nantes.fr/elnemo/). The third normal mode provided the major contribution to the transition between the two states. (B) Detailed view of the 2Fo-Fc electron density map contoured at 1σ around JAM-B (blue) and its surrounding GRASP55 PDZ1 (yellow) and PDZ2 (salmon) residues. (C) Detailed view of the 2Fo-Fc electron density map for the GRASP55 PDZ12/JAM-C complex. (D) LigPlot+ representation of the complex highlighting the hydrogen bonding interaction contacts (green) and the van der Waals and hydrophobic contacts (semi circles). Contact residues highlighted in red are shared in the JAM-B and JAM-C complex structures (see Fig 5C for comparison). (TIF) [file pgen.1006803.s009.tif]

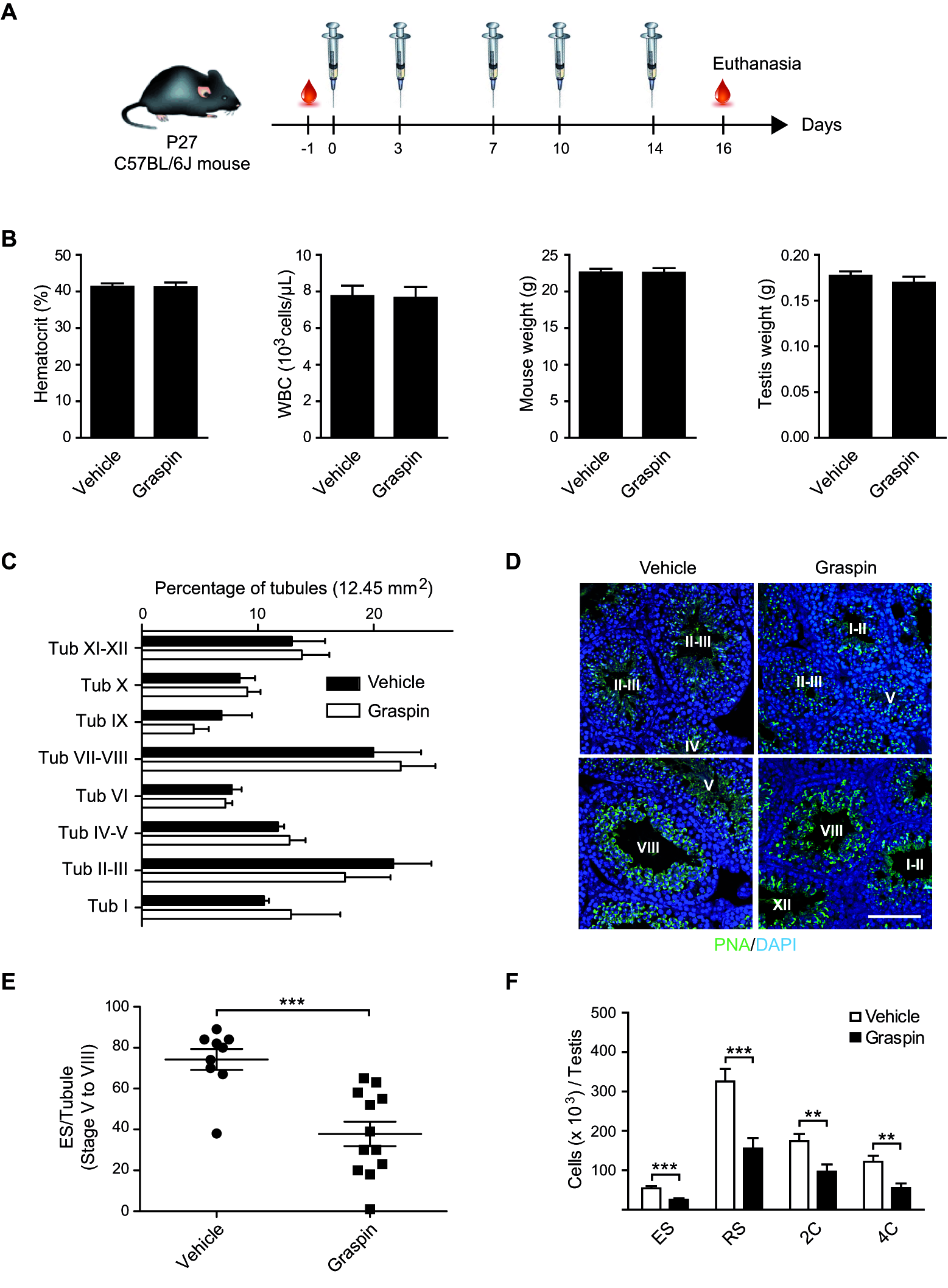

Supplement: S7 Fig — (A) Schematic representation of the protocol used for Graspin treatment. Graspin, dissolved in 10% DMSO, 90% corn oil, was injected in 27-day old C57BL/6J mice at 50 mg/kg on days 0, 3, 7, 10, and 14. Animals were sacrificed on day 16. Blood samples were collected the day before the first injection (day -1) and at endpoint (day 16). (B) Toxicity in Graspin-treated mice was evaluated by weight loss and hematological toxicity. No significant differences in weight, white blood cell count (WBC) or hematocrit were observed after two weeks of Graspin treatment. (C) Seminiferous tubule quantification at each stage from confocal pictures of mice treated with vehicle or Graspin. Staging was based on PNA staining as described in Nakata et al. (D) Representative confocal images of peanut agglutinin (PNA) and DAPI staining of seminiferous tubule sections used for quantification in (C). Seminiferous tubule stages are indicated. Scale bar, 100 μm. (E) Quantification of elongated spermatids (ES) per tubule (at stage V to VIII) of mice treated with vehicle or Graspin. Student’s unpaired t-test; ***: P<0.001. (F) Flow cytometry quantification of germ cell numbers isolated from testes of 27-days old mice treated with Graspin for two weeks and analyzed two days after the last Graspin injection. Vehicle, n = 10; Graspin, n = 10. Student’s unpaired t-test; **: P<0.01, ***: P<0.001. (TIF) [file pgen.1006803.s010.tif]

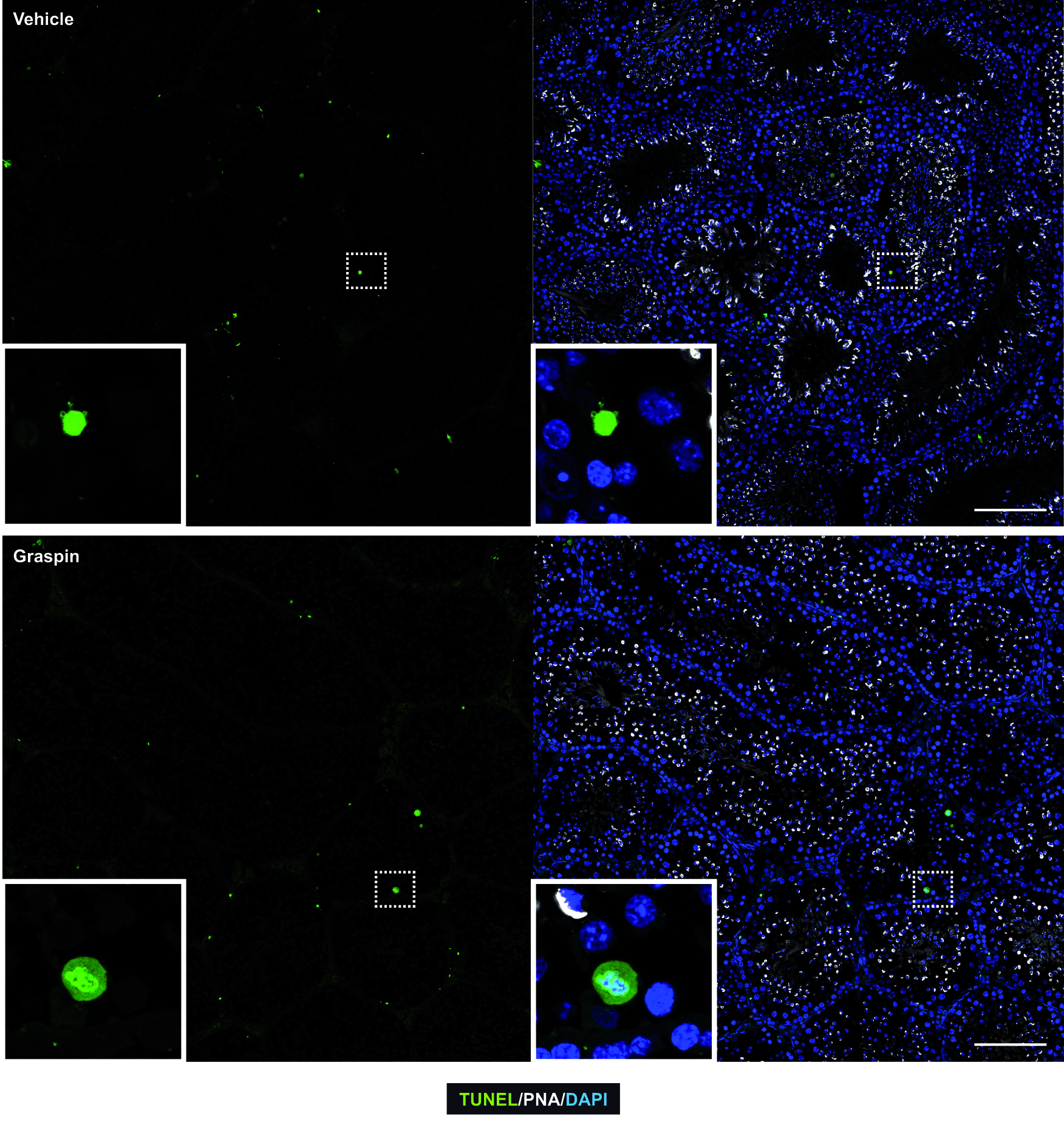

Supplement: S8 Fig — Representative large mosaic pictures (735 x 735μm) of TUNEL staining (green) obtained on testes sections from vehicle or Graspin treated mice. Rare cells are stained by TUNEL, indicating that Graspin does not induce apoptosis. Scale bar, 100 μm. (TIF) [file pgen.1006803.s011.tif]
