# Supplementary material for: Genetic, structural, and chemical insights into the dual function of GRASP55 in germ cell Golgi remodeling and JAM-C polarized localization during spermatogenesis
Source: PLoS Genet. 2017 Jun 15;13(6):e1006803. doi: 10.1371/journal.pgen.1006803 (PMC5472279; doi:10.1371/journal.pgen.1006803)
Supplement: S1 Text — (DOCX) [file pgen.1006803.s012.docx]

**Materials and Methods**

**Antibodies**

Rabbit anti-cKit (ref. 3074, Cell Signaling), rat anti-JAM-A H202.106 ([1](#_ENREF_1)), rabbit anti-JAM-B (pAb 829), rabbit anti-ZO-1 (ref. 61-7300, Zymed) and rabbit anti-SOX 9 (ref. AB5535, Millipore) primary antibodies were used in supplementary figures.

**Structural characterization and refinement**

All data were collected by the Shanghai Synchrotron Radiation Facility (SSRF) BL17U at 100 K. The data were processed and scaled with Xia2. All of the crystals were of space group I4_1_22 and contained two subunits per asymmetric unit, with a solvent content of approximately 72%. The structure was determined by molecular replacement using two separated PDZ domains of 3RLE as models. The solution was found using the Phaser program, and a partial model was constructed and improved after several iterative cycles of model building with Buccaneer and REFMAC. The model was then refined with Phenix. The final model of the GRASP55 PDZ domain with JAM-B was refined to 2.99 Å with an *R*_work_ of 22.5% and an *R*_free_ of 27.4% and contained GRASP55 residues 1 to 208 with 16 extra residues. Eight JAM-B peptide residues were found in one subunit, whereas six residues were found in the second one. The GRASP55 PDZ domain in complex with JAM-C was refined to 2.71 Å with an *R*_work_ of 25.6% and an *R*_free_ of 29.1%. This structure exhibited the same organization as the GRASP55/JAM-B complex, and five JAM-C peptide residues were found in each subunit. The accession codes are 5GMJ (complex of GRASP55 with JAM-B) and 5GMI (complex of GRASP55 with JAM-C).

***In silico* structure-based screening**

The *in-silico* screening was designed based on a dual strategy using molecular docking (Surflex Screen) and a Pharmacophoric filter (UNITY®), as implemented in Certara Tripos SYBYL-X2.0. The high-throughput molecular docking step was performed on the surface of GRASP55 extracted from the GRASP55/JAM-B complex structure using the Surflex Screen (default parameters) on the 143,218 commercially available compounds selected in our in-house 2P2I reference PPI-focused small compounds database ([2](#_ENREF_2)), which were treated and extended to 264,075 tautomers using Chemaxon 5.10. A maximum of 30 poses were generated by the docking algorithm for each compound, and the entire conformers database (approximately 7.7 M poses) was filtered out using the SYBYL UNITY® pharmacophoric filter derived from the GRASP55/JAM-B complex structure (Fig 4B). The filtered compounds were hierarchically classified and clustered using maximum common substructures containing at least 12 atoms (LibMCS tool from Chemaxon with all other settings as defaults). One representative compound exhibiting the lowest molecular weight was selected from each cluster. The 49 selected compounds were purchased and tested experimentally by HTRF.

**Generation of *Gorasp2* knock-out mice**

*Gorasp2^-/-^* mice were generated by deleting the *Gorasp2* coding sequence, including exon 3 to exon 6 (GenOway, Lyon, France). Targeting vector was obtained by successive cloning of PCR products. Briefly, the Sv129/Pas mouse genomic DNA was amplified to generate the different fragments. The 5’distal part of long homology arm containing exon 2 (3.7kb) and the 5’ proximal part of long homology arm (3,5kb) were inserted in 5‘ and 3’ of a LoxP containing vector using respectively PflMI/XbaI and NheI/HpaI digestions. The 3’ short homology arm (3,3 kb) was inserted in 3’of a Frt/Neomycin/Frt/LoxP containing vector using HpaI/KpnI digestion. The Frt/Neomycin/Frt/LoxP/3’ short homology fragment was then digested by SmaI/SexA1 and inserted in 3’of the 5’ long homology arm containing LoxP sequence. Finally, the DTA cassette was added 5’ to Exon 2 to generate the targeting vector (see in S1 Figure). The targeting vector containing LoxP sites flanking exon 3 to exon 6, neomycin-positive selection cassette (NeoR) and diphtheria toxin A-negative selection cassette (DTA) was electroporated into 129 Sv/Sav embryonic stem (ES) cells. Recombined ES cells were selected using the G418 antibiotic. Southern blot and PCR screening confirmed the homologous recombination event (selected primers for PCR: 5'-GTGAGACGTGCTACTTCCATTTGTCACG-3' and 5'-GTAATGACAGAGAAAGAGCTTGAAGCAACAAC-3'; selected primers for Southern blot analysis: 5'-TGAAGTTGGATACTGCCCACTCCACC-3' and 5'-TGGCTCACTTCTTCCTCTTCCTCTGG-3'). Validated clones were selected for C57BL/6 blastocyst injection and generation of chimeric animals ([3](#_ENREF_3)). A *Gorasp2* constitutive knock-out model was obtained by breeding chimeras with CMV-Cre deleter mice, resulting in excision of the LoxP-flanked sequence. Cre deletion causes the deletion of the 503 bp *Gorasp2* coding sequences and frame shift resulted in a premature stop codon. Mice used in this study were backcrossed onto the C57BL/6J strain for at least six generations.

**Genotyping of *Gorasp2^-/-^* mice**

Genotyping was achieved by PCR of genomic DNA extracted from mouse tails. The PCR primers were as follows: 5'-GCTTCTCGGTTGATTCTGAAGGGAGC-3' (52213); 5'-CTCTAGGAAGCAAGGAAACCATACATTGG-3' (52194) and 5'-CATAGCCTCAGAATTGCCCTTAGAGTCG-3' (52193). DNA was amplified using Platinum Taq DNA Polymerase (Invitrogen) with the following cycling procedure: 94°C for 2 min, 30 cycles of 30 s at 94°C, 30 s at 55°C, and 1 min at 68°C, and a final extension step at 68°C. The resulting 544-bp fragment for the WT allele and 754-bp fragment for the KO allele were both obtained using heterozygous mice.

**Testis Golgi area and shape quantification**

Golgi were detected from GM130 testis staining. Area and shape quantification were performed using the method described in Fig A of S4 Figure. Shape factor close to one is associated with circular objects, with major axis equal to minor axis. Medians were calculated and depicted by dashed lines. The histogram of Golgi areas presents a bimodal distribution with structures either larger or smaller than 7.7 µm^2^. We thus represented Golgi with the following color code for a better visualization: from blue to green for small structures, below 7.7 µm^2^ and from yellow to red for larger ones. Quantification was performed from mosaic images acquired using an LSM 510 META confocal microscope (Zeiss), representative of around 30 testis tubule sections.

**MEFs Golgi density quantification**

MEFs cultured on glass coverslips coated with fibronectin (5 µg/mL) and gelatin (0.1%), were fixed and permeabilized in methanol -20°C during 5 min and blocked in PBS 1% BSA during 1 h at room temperature. Primary antibodies and secondary antibodies were incubated successively for 1h in a humidified chamber at room temperature. Images were acquired using an LSM 510 META confocal microscope (Zeiss). Image analysis was done using home-made Matlab script to perform the four steps described in Fig B of S4 Figure and some Golgi density scores were illustrated in Fig C of S4 Figure. Around 20 to 100 Golgi are analyzed per condition for each experiment. Results represent a pool of at least three independent experiments.

**Hematoxylin-eosin-safran staining**

Testes were fixed in 1% paraformaldehyde for 4 h at room temperature and enclosed in cassettes. Tissues were prepared for paraffin infiltration using the automated tissue processor ASP 300 (Leica) in 3 steps: the dehydration step comprised successive immersions in absolute ethanol with three 30-min baths followed by three 1-h baths at 37°C; the clarification step comprised successive immersions in three 1-h baths in Histolemon at 37°C; the final infiltration step comprised successive immersions in paraffin baths for 90 min at 59°C and 150 min and 210 min at 58°C. The tissues were manually placed in metallic molds containing liquid paraffin and incubated at room temperature until solidification. Serial sections (5-µm-thick) were cut with an electronic rotary microtome, HM 340E (Thermo Scientific), and dried for 1 h at 56°C. HES staining was performed using the automated slide stainer JUNG XL (Leica). The slides were dehydrated by four successive 2-min baths in absolute ethanol followed by two successive 2-min baths in Histolemon and were then mounted in Pertex medium using the automated glass coverslipper CV5030 (Leica). The slides were scanned using NanoZoomer 2.0-HT digital slide scanner (Hamamatsu).

**Blood analysis**

Red and white blood cells from mouse blood were quantified using the IDEXX ProCyte Dx® Hematology Analyzer (IDEXX Laboratories).

**Supplemental References**

1. Malergue F, Galland F, Martin F, Mansuelle P, Aurrand-Lions M, Naquet P. A novel immunoglobulin superfamily junctional molecule expressed by antigen presenting cells, endothelial cells and platelets. Mol Immunol. 1998 Dec;35(17):1111-9.

2. Hamon V, Bourgeas R, Ducrot P, Theret I, Xuereb L, Basse MJ, et al. 2P2I HUNTER: a tool for filtering orthosteric protein-protein interaction modulators via a dedicated support vector machine. J R Soc Interface. 2014 Jan 6;11(90):20130860.

3. Capecchi MR. Gene targeting in mice: functional analysis of the mammalian genome for the twenty-first century. Nat Rev Genet. 2005 Jun;6(6):507-12.
